# Assessment of anti-CD20 antibody pre-treatment for augmentation of CAR-T cell therapy in SIV-infected rhesus macaques

**DOI:** 10.3389/fimmu.2023.1101446

**Published:** 2023-02-07

**Authors:** Mary S. Pampusch, Emily N. Sevcik, Zoe E. Quinn, Brianna C. Davey, James M. Berg, Ian Gorrell-Brown, Hadia M. Abdelaal, Eva G. Rakasz, Aaron Rendahl, Pamela J. Skinner

**Affiliations:** ^1^ Department of Veterinary and Biomedical Sciences, University of Minnesota, St. Paul, MN, United States; ^2^ Wisconsin National Primate Research Center, University of Wisconsin, Madison WI, United States

**Keywords:** CAR-T cell, CXCR5, anti-CD20, SIV, HIV, rhesus macaque

## Abstract

During chronic HIV and SIV infections, the majority of viral replication occurs within lymphoid follicles. In a pilot study, infusion of SIV-specific CD4-MBL-CAR-T cells expressing the follicular homing receptor, CXCR5, led to follicular localization of the cells and a reduction in SIV viral loads in rhesus macaques. However, the CAR-T cells failed to persist. We hypothesized that temporary disruption of follicles would create space for CAR-T cell engraftment and lead to increased abundance and persistence of CAR-T cells. In this study we treated SIV-infected rhesus macaques with CAR-T cells and preconditioned one set with anti-CD20 antibody to disrupt the follicles. We evaluated CAR-T cell abundance and persistence in four groups of SIVmac239-infected and ART-suppressed animals: untreated, CAR-T cell treated, CD20 depleted, and CD20 depleted/CAR-T cell treated. In the depletion study, anti-CD20 was infused one week prior to CAR-T infusion and cessation of ART. Anti-CD20 antibody treatment led to temporary depletion of CD20+ cells in blood and partial depletion in lymph nodes. In this dose escalation study, there was no impact of CAR-T cell infusion on SIV viral load. However, in both the depleted and non-depleted animals, CAR-T cells accumulated in and around lymphoid follicles and were Ki67+. CAR-T cells increased in number in follicles from 2 to 6 days post-treatment, with a median 15.2-fold increase in follicular CAR-T cell numbers in depleted/CAR-T treated animals compared to an 8.1-fold increase in non-depleted CAR-T treated animals. The increase in CAR T cells in depleted animals was associated with a prolonged elevation of serum IL-6 levels and a rapid loss of detectable CAR-T cells. Taken together, these data suggest that CAR-T cells likely expanded to a greater extent in depleted/CAR-T cell treated animals. Further studies are needed to elucidate mechanisms mediating the rapid loss of CAR-T cells and to evaluate strategies to improve engraftment and persistence of HIV-specific CAR-T cells. The potential for an inflammatory cytokine response appears to be enhanced with anti-CD20 antibody treatment and future studies may require CRS control strategies. These studies provide important insights into cellular immunotherapy and suggest future studies for improved outcomes.

## Introduction

Worldwide, over 38 million people were living with HIV in the past year (1). Antiretroviral drugs are effective in reducing virus levels in these patients, often to undetectable levels; however, the drugs are incapable of fully eliminating the cellular reservoir of the virus (2–4). Successful treatment of HIV relies on lifelong adherence to ART which may be difficult or impossible for patients with limited access to healthcare. To improve the wellbeing of people living with HIV, alternative strategies for elimination of the virus have been of intense interest. During chronic HIV or SIV infection, viral replication occurs predominantly within the B cell follicles of secondary lymphoid tissues (5, 6). Interestingly, within SIV elite controllers, there is some productive infection in T follicular helper cells inside the follicle but none outside the follicle, highlighting the immune privileged nature of B cell follicles (7). Typically, virus-specific CD8+ T cells are found at low levels in the B cell follicle (5, 6, 8, 9) and have been suggested to have reduced cytolytic activity (10), likely leading to a failure to effectively eliminate the virally infected cells. Increasing the number of follicular virus-specific cytotoxic T cells and overcoming the barrier of the B cell follicle are critical areas of interest towards HIV cure strategies.

Chimeric antigen receptor (CAR) T cells have shown promise in the treatment of B cell leukemias and lymphomas with two products now in clinical use (11–13). CAR-T cells are also of particular interest in the treatment of HIV since they can be constructed to specifically bind to, and eliminate, virally infected cells (14). In this study, we used a CAR designed to specifically target cells infected with simian immunodeficiency virus (SIV), a primate model for HIV. The CAR contains domains of CD4 (domains 1 and 2) as well as the carbohydrate recognition domain of mannose binding lectin (MBL) to facilitate binding to the viral glycoprotein, gp120 (15, 16). In order to target the CAR-T cells to the B cell follicle, the CAR construct also contains the chemokine receptor, CXCR5 (17–19), which directs the cells to the follicular chemokine CXCL13 (20, 21). Previous studies have demonstrated that the CAR/CXCR5 T cells homed to the B cell follicle and were potentially efficacious in control of levels of SIV after ART release (22). However, the cells failed to persist long-term in the treated animals potentially due to the absence of a lymphodepletion regimen prior to infusion of the CAR/CXCR5-T cells.

Most CAR-T cell immunotherapies include a lymphodepletion pre-treatment such as cyclophosphamide to improve engraftment and persistence of the CAR-T cells (23). Cyclophosphamide chemotherapy targets and kills dividing cells, and reduces the number of T, B, and NK cells. This reduction aids CAR-T cell expansion by creating space for homeostatic proliferation, by removal of immunosuppressive cells, and by removal of cytokine sinks leading to greater availability of IL-7 and IL-15 survival cytokines (24–27). If successful, the preconditioning may lead to enhanced expansion of the CAR-T cells; however, expansion of CAR-T cells is linked to an increased risk of cytokine release syndrome (CRS) (28, 29). Additionally, cyclophosphamide pre-treatment is quite harsh and may lead to cytopenias, mucosal barrier impacts, and the progression of pre-existing infections (30), which could be particularly concerning for people living with HIV. Immunomodulatory and/or lymphodepleting agents are showing promising results in increasing the efficacy of CAR-T cells directed towards solid tumors. These agents alter the immunosuppressive microenvironment of certain tumors where T cells may be dysfunctional or physically excluded (31–33). In a similar spirit, disruption of immune-privileged B cell follicles in HIV has been suggested as a potential approach to increase access of virus-specific cytotoxic T cells to the follicles and augment HIV cure strategies (34–36).

To evaluate a milder lymphodepletion pre-treatment that would also disrupt B cell follicles and potentially improve the efficacy of CAR-T cell immunotherapy, we examined the impact of pre-treatment with anti-CD20 antibody on the expansion and persistence of CD4-MBL-CAR/CXCR5-T cells in rhesus macaques. Anti-CD20 antibody treatment leads to interaction with, and elimination of, CD20+ B cells *via* complement-dependent cytotoxicity, antibody-dependent cellular cytotoxicity, antibody dependent cellular phagocytosis, and direct apoptosis (37, 38) leading to a loss of follicular structure in secondary lymphoid organs (39). All B cell subsets are not equally impacted by anti-CD20 antibody treatment. CD20 expression is limited; it is expressed from the pre-B cell to differentiated B cell stages but is not expressed on plasma cells (40). In a study of the impact of rituximab on B cell subsets in lymph node biopsies, the anti-CD20 drug significantly depleted naive B cells, unswitched memory B cells, and follicular B cells but did not have an impact on isotype- switched memory B cells (41). CD20+ B cell depleted monkeys have impaired humoral immune responses but can still generate cellular immune responses (42). Previous primate studies used anti-CD20 antibody doses of 20-50 mg/kg, usually with multiple administrations, which had prolonged impacts on CD20+ cells in blood and lymphoid tissues (42–44). One study used a single 5 mg/kg dose in rhesus macaques (45) and found a temporary depletion of CD20+ cells in the inguinal lymph node with recovery by 21 days post-depletion. We chose to similarly administer a single low dose (7 mg/kg) of anti-CD20 7 days prior to CAR-T cell infusion in order to facilitate a temporary depletion of the B cell follicles. We hypothesized that temporary CD20+ B cell disruption in the follicles would create space for CAR-T cells to proliferate and allow CAR-T cells to persist in the newly formed follicles to control follicular viral replication. In this study, we evaluated the impact of CD4-MBL-CAR/CXCR5 T cell infusion into rhesus macaques that either had no lymphodepletion pre-treatment or were pre-treated with anti-CD20 antibody. Similar to our previous study, we found that the CD4-MBL-CAR/CXCR5 T cell therapy was safe and showed promise for sustained remission of SIV. With the addition of anti-CD20 pre-treatment, we found that the CAR-T cells accumulated in and around lymphoid follicles, proliferated and expanded the first week, then abruptly disappeared prior to the re-emergence of SIV. The combination of CD20 depletion and CAR-T cell treatment was also associated with increased and prolonged IL-6 expression. Overall, a single low dose anti-CD20 pre-treatment may effectively enhance the proliferation and accumulation of CAR-T cells at viral reservoir sites, but this treatment requires inclusion of IL-6 inhibitors to prevent CRS, and further optimization of CAR-T cells to promote long-term survival following *in vivo* proliferation.

## Materials and methods

### Animal study design

Rhesus macaques that were negative for the class I alleles Mamu-B*008 and Mamu-B*017:01 were used in these studies. PBMCs were collected from the animals by density gradient centrifugation from blood draws prior to infection. The cells were cryopreserved in CryoStor CS5 (BioLife Solutions Inc.) at a concentration of 8-19 million cells/ml and transported and stored in liquid nitrogen until use. All animals were infected intrarectally with SIVmac239 (1000-3000 TCID_50_) for 30-75 days prior to the initiation of ART consisting of 5.1 mg/kg Tenofovir Disoproxil Fumarate (TDF) (Gilead), 40 mg/kg Emtricitabine (FTC) (Gilead) and 2.5 mg/kg Dolutegravir (DTG) (Viiv). ART was continued until the day of cell infusion with the exception of two of the depleted animals, R09072 and R16041, which had a short ART interruption at days 88-97 post-infection. Blood samples were drawn biweekly to monitor viral loads, and all animals had undetectable viral loads for at least 4 weeks prior to infusion. The untreated and CAR-T groups of animals did not undergo lymphodepletion. Seven days prior to infusion of CAR-T cells, the CD20-depleted animals were treated intravenously with a dose of 7 mg/kg of a rhesus IgG1 recombinant Anti-CD20 (2B8R1) monoclonal antibody which was engineered and produced by the Nonhuman Primate Reagent Resource (NIH Nonhuman Primate Reagent Resource Cat# PR-2287, RRID : AB_2716323). All CD20-depleted, CAR-T cell treated animals, with the exception of R09072, were dosed with 11 mg/kg of Siltuximab (anti-IL-6) on the day of cell infusion to reduce the possibility of cytokine release syndrome (CRS). All CD20-depleted animals, with the exceptions of deceased animals R09072, R11025 and R16041, were treated with 3 mg/kg rhesus recombinant anti PD-1 obtained from the Nonhuman Primate Reagent Resource (AB_2819337) at 44-79 days post infusion in order to potentially reverse T cell exhaustion (**Table 1**).

**Table 1:** Animal information.

Group	Animal	Age (yr)	Gender	Weight (kg)	Peak VL post-infusion (x 10^8^)	Pre-infection CD4 count	ART (days)	anti-IL-6 (DPT)	anti-PD-1 (DPT)	Necropsy (DPT)
CAR-T	R16002	3.8	F	5.3	0.31	NA	237	NA	NA	231
CAR-T	Rh2905	8.6	F	5.1	2.02	890	181	NA	NA	260
CAR-T	R14069	5.3	M	7.7	0.66	452	161	NA	NA	210
CAR-T	R16084	5.2	M	6.0	0.17	799	117	NA	NA	181
Untreated	R12034	7.5	F	5.3	0.56	NA	237	NA	NA	231
Untreated	Rh2918	3.5	M	6.0	0.86	719	181	NA	NA	251
Untreated	RhBK73	5.2	M	7.7	0.64	748	161	NA	NA	190
Untreated	R13036	6.5	F	6.5	0.82	1153	117	NA	NA	176
Depleted/CAR-T	R09072	11.6	F	6.5	0.46	2005	264	NA	NA	2
Depleted/CAR-T	Rh3027	4.2	M	8.4	1.38	1571	145	0	77	107
Depleted/CAR-T	Rh2997	4.2	M	8.4	0.33	2652	173	0	65	83
Depleted/CAR-T	Rh2783	13.8	F	8.1	1.30	796	334	0	44	84
Depleted	R16041	4.8	M	8.3	0.50	1509	264	NA	NA	45
Depleted	Rh3024	4.0	M	8.1	0.39	2398	145	NA	77	107
Depleted	Rh3003	4.2	M	9.6	0.29	1303	173	NA	65	86
Depleted	R11025	9.8	F	8.4	1.51	977	334	NA	NA	42

NA, not administered.

### Cell manufacturing and infusion

The CD4-MBL-CAR/CXCR5 construct is a bi-specific CAR which contains rhesus codon-optimized CD4 and MBL domains, leading to specificity for SIV, linked to extracellular hinge, transmembrane and co-stimulatory domains of rhesus CD28 and the CD3 zeta activation domain (15). The follicular homing receptor, CXCR5, is linked *via* a self-cleaving peptide, P2A (16). The genes were subcloned into the pMSGV1 gammaretroviral vector and gammaretroviruses were produced by lipofectamine-mediated transfection of 293T cells. CD4-MBL CAR/CXCR5-T cells were manufactured using the CD4-MBL CAR/CXCR5 gammaretrovirus as outlined previously (46, 47). Briefly, the PBMCs were thawed and stimulated with plate-bound anti-CD3 (clone FN18) and soluble anti-CD28 (clone 28.2) for two days prior to retronectin-mediated transduction with gammaretroviral vector at an MOI of 0.5. Two days after transduction, the cells were placed in G-Rex 6 well plates (Wilson Wolf Corporation) and expanded for 4 days. All media contained 50 U/ml IL-2. Prior to infusion, cells were collected, washed, and resuspended at a density of 2 × 10^7^ cells/mL in PBS containing 10% autologous serum, packed on ice and transported to the Wisconsin National Primate Research Center (WNPRC). The T cell products were infused intravenously over 20 min while the animals were sedated. A veterinarian was present during the entire infusion. The dose of cells ranged from 0.1 to 2.3 × 10^8^ cells/kg (**Table 2**). Following infusion, the staff of the Animal Services Unit observed the animals at least twice daily and evaluated the animals for signs of pain, illness, and stress by observing appetite, stool, typical behavior, and physical condition. The weight of the animals was monitored routinely throughout the protocol.

### Tissue, blood, and cell collection

Blood samples were drawn for viral load determination immediately before and after infusion and on days 2, 6, 10, 14 and then biweekly until necropsy. Complete blood counts (CBC) were monitored biweekly throughout the experiment. Lymph node biopsies and BAL samples were collected on days 2, 6, 14, 28 and 56 post-infusion. Disaggregated lymph node cells and PBMCs were isolated at the University of Wisconsin. Animals were necropsied between day 2 and 260 post-infusion (**Table 1**).

### Viral load determination

Viral loads were measured by Virology Services (WNPRC). vRNA was isolated from plasma samples using the Maxwell Viral Total Nucleic Acid Purification kit on the Maxwell 48RSC instrument (Promega, Madison WI). vRNA was then quantified using a highly sensitive qRT-PCR assay based on the one developed by Cline et al. (48). RNA was reverse transcribed and amplified using TaqMan Fast Virus 1-Step Master Mix (Invitrogen) on the LightCycler 480 or LC96 instrument (Roche, Indianapolis, IN) and quantified by interpolation onto a standard curve made up of serial ten-fold dilutions of *in vitro* transcribed RNA. RNA for this standard curve was transcribed from the p239gag_Lifson plasmid, kindly provided by Dr. Jeffrey Lifson, (NCI/Leidos). The final reaction mixtures contained 150 ng random primers (Promega, Madison, WI), 600 nM each primer and 100 nM probe. Primer and probe sequences are as follows: forward primer: 5′- GTCTGCGTCATCTGGTGCATTC-3′, reverse primer: 5′-CACTAGCTGTCTCTGCACTATGTGTTTTG-3′ and probe: 5′-6-carboxyfluorescein-CTTCCTCAGTGTGTTTCACTTTCTCTTCTGCG-BHQ1-3′. The reactions cycled with the following conditions: 50°C for 5 min, 95°C for 20 s followed by 50 cycles of 95°C for 15 s and 62°C for 1 min. The limit of detection of this assay is 100 copies/mL.

### Flow cytometry

Multiparametric flow cytometry was performed on fresh, transduced PBMCs and on thawed PBMCs or disaggregated lymph node cells collected post-infusion. Cells were incubated with Live/Dead Near-IR (Invitrogen); Alexa Fluor 700 or Brilliant Violet 421 mouse anti-human CD3 (SP34-2), FITC or Brilliant Violet 650 mouse anti-human CD4 (M-T477), Brilliant Violet 510 or Brilliant Violet 786 mouse anti-human CD8 (RPA-T8), PerCP/Cy5.5 mouse anti-human CD95 (DX2), Brilliant Violet 605 mouse anti-human CD28 (28.2), PE-CF594 mouse anti-human CCR7 (150503), Brilliant Violet 510 mouse anti-human CD45RA (5H9), PE-Cy7 or Brilliant Violet 650 mouse anti-human CD20 (2H7) (BD Biosciences); Phycoerythrin (PE) or PE-Cyanine7 mouse anti-human CXCR5 (MU5UBEE) (eBiosciences); MBL (3E7) (Invitrogen) conjugated to Alexa Fluor 647. To detect SIV-specific CD8^+^ T cells, samples were incubated with PE-labeled GAG-CM9 (NIH Tetramer Core) at 37°C for 15 min prior to antibody staining. Samples were run on a CytoFLEX flow cytometer (Beckman) and analyzed with FlowJo software v10.7.1 (BD Life Sciences).

### Luminex assay

Serum samples were stored at -80°C prior to analysis. Samples were tested by the Cytokine Reference Laboratory (University of Minnesota) using the magnetic bead set PRCYTOMAG-40K (EMD Millipore). Samples were analyzed for Non-Human Primate (NHP)-specific TNFα, IFNγ, IL-6, IL-1β and IL-2 using the Luminex platform and performed as a multi-plex. Fluorescent color-coded beads coated with a specific capture antibody were added to each sample. After incubation and washing, biotinylated detection antibody was added followed by phycoerythrin-conjugated streptavidin. The beads were read on a Luminex instrument (Bioplex 200). Samples were run in duplicate and values were interpolated from five-parameter fitted standard curves.

### RNAscope *in situ* hybridization and immunofluorescence

The RNAscope multiplex fluorescent kit V2 (Advanced Cell Diagnostics) was used to detect CAR/CXCR5-transduced cells and SIV vRNA as described previously (22). Briefly, 5 µm FFPE sections were deparaffinized, treated with hydrogen peroxide, boiled in RNAscope target retrieval reagents, treated with proteinase, and then incubated with probes at 40°C overnight. For singleplex detection of transduced CAR/CXCR5 T cells, a custom probe was used in channel C1. For duplex detection of CAR/CXCR5 T cells and SIV vRNA, SIVmac239 C2 no env antisense probe was diluted 1:50 in the custom CAR/CXCR5 C1 probe. Sections were washed in 0.5x RNAscope wash buffer followed by amplification and C1 development and, for SIV vRNA detection, C2 development. Opal 570 was used for C1, and Opal 690 was used for C2. Following RNAscope, sections were washed in TBS with 0.05% Tween 20, blocked with TBS with 10% normal goat serum and 1% BSA, and stained with 1:100 mouse-anti-human CD20 (Clone L26, Biocare) followed by Opal Polymer HRP Ms + Rb and then Opal 520. Alternately, sections were stained with 1:100 mouse anti-human CD20 with 1:200 rabbit anti-Ki67 (Clone SP6, ThermoFisher Scientific), followed by Alexa Fluor 488 goat anti-mouse and Cy5 goat anti-rabbit.

To stain sections with anti-CD20 and/or anti-Ki67 antibodies only, sections were deparaffinized, boiled in citrate buffer (pH 6), washed in TBS with 0.05% Tween 20, blocked with TBS with 10% normal goat serum and 1% BSA, and stained with mouse anti-human CD20 and rabbit anti-Ki67, followed by Alexa Fluor 488 goat anti-mouse and Cy5 goat anti-rabbit. To stain sections with anti-CXCL13 and anti-CD79a, sections were deparaffinized, boiled in Tris-EDTA buffer (pH 9), blocked with 10% donkey serum in TBS, and stained with 1:75 goat anti-human CXCL13 (R&D Systems, AF801) and 1:50 mouse anti-CD79a (Clone HM47, Santa Cruz Biotechnology), followed by Alexa Fluor 488 donkey anti-goat and Alexa Fluor 555 donkey anti-mouse.

### Imaging and analysis

Sections were imaged using a Leica TCS SPE DM6000 confocal microscope. Montage images of tissue sections were used for analysis. Leica application suite software was used to delineate and measure follicular and extrafollicular areas, count CAR/CXCR5 T cells and SIV vRNA+ cells and identify Ki67+ cells. Areas of highly clustered CD20+ cells were identified as B cell follicles. Areas of highly clustered Ki67+ cells were identified as germinal centers (GCs).

To determine concentrations of CAR/CXCR5 T cells over time, we examined 2 to 3 tissue sections, with at least 6 follicles per animal for each analyzed time point. Each biopsy collected a single lymph node for *in situ* analysis, therefore, all analyzed sections at each time point come from a single anatomical site. A median of 4.64 mm^2^ (range 0.28-6.24 mm^2^) of follicular area was analyzed. 279 follicles were analyzed with a median of 35 (range 6-53) per animal. A median of 13.84 mm^2^ (range 0.79-19.25 mm^2^) of extrafollicular area was analyzed per animal. 182 extrafollicular areas were analyzed with a median of 22 (range 4-36) per animal. Concentrations of SIV vRNA+ cells were determined for the 28 DPT (days post-treatment) time point. We examined 2 to 3 tissue sections, with at least 6 follicles per animal. A median of 1.00 mm^2^ of follicular area (range 0.49-1.62 mm^2^) per animal was analyzed. 116 follicles were analyzed, with a median of 7.5 follicles was analyzed per animal (range 6-13). A median of 3.42 mm^2^ of extrafollicular area (range 0.72-7.40 mm^2^) was analyzed per animal. 82 extrafollicular areas were analyzed, with a median of 5 extrafollicular areas per animal (range 2-13).

### Anti-drug antibody detection

The method used to detect anti-CAR antibodies was previously outlined (49). In brief, cryopreserved transduced CAR-T cells, as well as mock transduced PBMCs, were thawed and used as antibody binding targets. Serum from treatment animals was heat inactivated for 30-35 minutes at 56°C, diluted five-fold in PBS, and added to wells containing 1x10^5^ CAR or mock transduced T cells. After a 20 min incubation at 4° C, the cells were washed twice and stained for 20 minutes with live/dead NIR, anti-human CD3 (SP34-2), anti-human CD4 (M-T477), anti-human MBL (3E7), and anti-human IgG (G18-145), then fixed with 1% paraformaldehyde, captured on a CytoFLEX flow cytometer (Beckman) and analyzed with FlowJo software v10.7.1 (BD Life Sciences).

### Statistical analysis

All statistical analysis was performed using GraphPad Prism v 9.3.1. Comparisons between groups utilized the nonparametric Mann Whitney test.

## Results

### Study animals

The response to CAR/CXCR5 T cells was studied in SIVmac239-infected ART-suppressed rhesus macaques that had no lymphodepletion pre-treatment (CAR-T) and in animals that were pre-treated with 7 mg/kg anti-CD20 antibody 7 days prior to cell infusion (depleted/CAR-T). Each treated animal was paired with a control animal. CAR-T animals were paired with untreated control animals, and depleted/CAR-T animals were paired with CD20-depleted animals (depleted). The age, weight, peak viral loads and CD4 frequencies for all animals are presented in **Table 1**. Additionally, the table presents the length of time on ART, whether and when they were treated with anti-IL-6 or anti-PD-1 antibodies, and the length of time in the study. All animals were negative for the protective alleles Mamu-B*008 and Mamu-B*017.01.

### Impact of CAR-T cell infusion on SIV mac239-infected rhesus macaques

**Figure 1:**
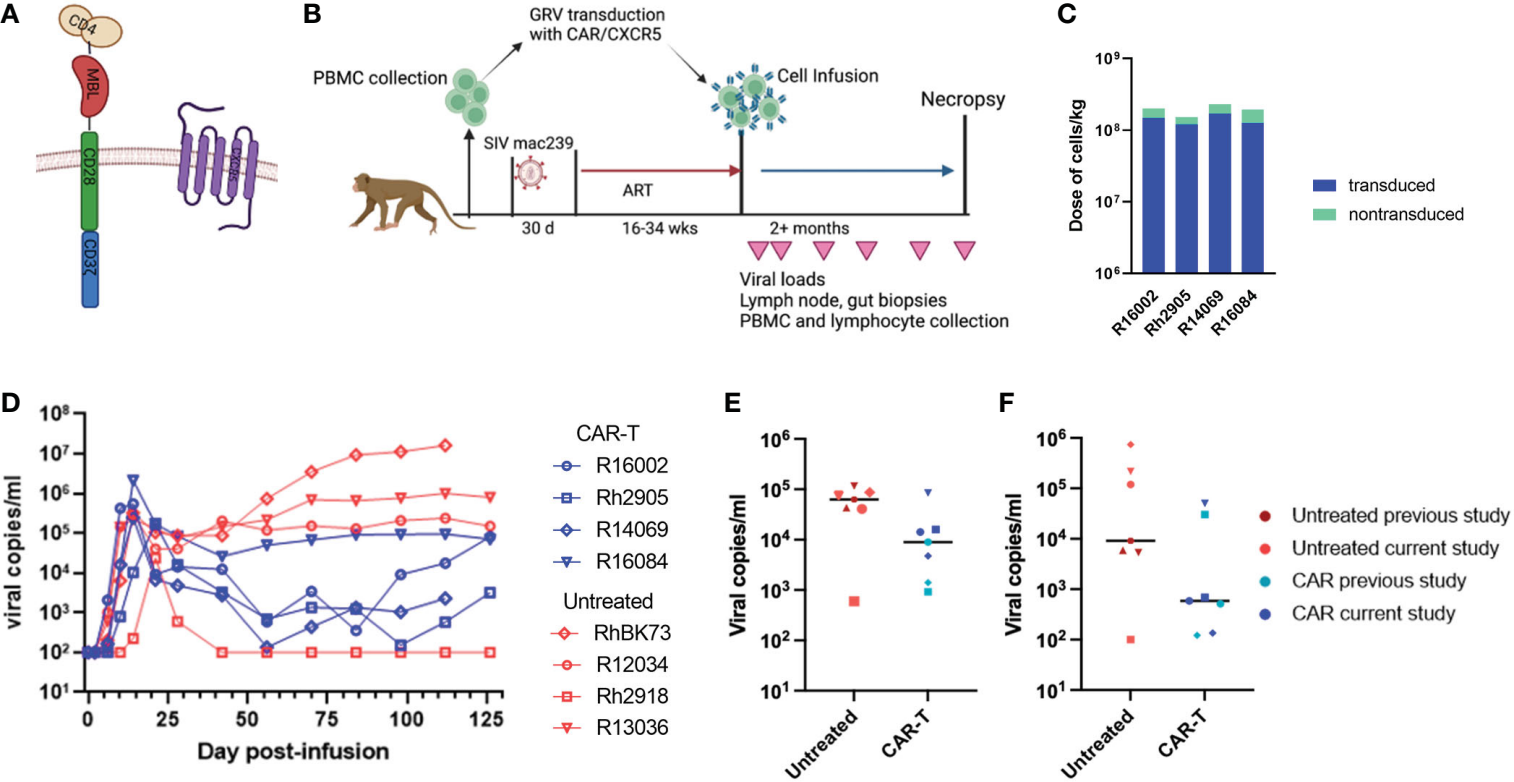
CAR/CXCR5 T cell study without B cell depletion. **(A)** The CAR contains CD4 D1/D2 domains, the carbohydrate recognition domain of MBL, CD28 transmembrane and co-stimulatory domains and a CD3 zeta signaling domain; CXCR5 is co-expressed with the CAR. **(B)** The study design. **(C)** The total number of cells infused into the treatment animals with the transduced cells presented in blue and non-transduced in green. **(D)** Viral copies per ml over time in the untreated (red) and CAR-T treated (blue) animals. **(E)** Combined data showing viral copies/ml at 28 DPT or **(F)** viral copies/ml at 56 DPT in untreated (maroon or red) and CAR-T treated (blue or teal) animals from this study and a previously published study. Each point represents one animal. The lines in **(E, F)** represent the median viral load for each group. **(A, B)** were created with BioRender.com.

Our initial study expanded on a previous promising pilot study in which three ART suppressed SIVmac251 infected rhesus macaques were treated with CD4-MBL CAR/CXCR5 T cells (CAR-T) and showed reductions in viral loads compared to three untreated control animals (22). Similar to the pilot study, here we treated 4 SIVmac239 infected ART-suppressed rhesus macaques with CAR-T cells on the day of ART release. The CAR construct and study design are outlined in **Figures 1A, B**. A high percentage of the cells were transduced and co-expressed both the CAR and CXCR5 proteins (median 74%; range 66-78%) (**Table 2**; **Supplementary Figure 1A**). While the cell production protocol is identical for each transduction and expansion, there is some animal-to-animal variability in the resultant CAR-T cell products. The cell products included populations with a central memory phenotype (median 66%; range 44-80%), with most being CCR7+ with a range of 56-79% (**Table 2**). The majority of cells were negative for the apoptosis marker annexin, however 10-23% of the cells were annexin positive (**Table 2**). Animals were treated with 1.5 to 2.3 x 10^8^ cells/kg (**Figure 1C**; **Table 2**). We previously found these doses of cells to be safe in rhesus macaques, leading to high levels of CAR/CXCR5-T cells in follicles (22). Plasma viral loads were monitored throughout the study (**Figure 1D**). With the exception of one of the untreated animals that spontaneously controlled viral loads below the level of detection, all other untreated animals had viral loads that were higher than those of the CAR-T treated animals for the duration of the study. We further examined the viral loads at 28 and 56 DPT by combining the current data with control and CAR-T treated animal viral loads presented in our previous pilot study (22). In **Figures 1E, F**, the untreated animals are presented in red (current study) or maroon (previous pilot study) and the CAR-T treated animals are presented in blue (current study) or teal (previous pilot study). With the combined studies, we did not find a significant difference between CAR-T and untreated groups at 28 (p=0.097) or 56 (p=0.18) DPT (**Figures 1E, F**). Likewise, no differences in groups were detected with regard to time to viral rebound after release from ART, or in area under the curve from infusion to 28 DPT or day of infusion to 56 DPT (data not shown). Although at this time, there is not enough evidence to detect a difference between groups, we cannot rule out the possibility that this lack of statistical significance is due to a low animal number in the studies. Importantly, we observed that 71% (5/7) of CAR-T cell treated animals compared to only 14% (1/7) of untreated animals had plasma viral loads below 1000 virus copies/ml two months after ART release and CAR-T cell infusion (**Figure 1F**). While some of the animals in these cohorts were sacrificed shortly after 60 days post-treatment, a number of animals were maintained for longer periods. These data show that animals that were controlling infection at 2 months-post-treatment largely maintained control of infection for the additional 3 months that they were monitored (**Supplementary Figure 2**).

**Table 2:** CAR-T cell information.

Group	Animal	Cells/kg infused	%MBL+/ CXCR5+	CAR cells/kg	CD4+/CD8+/CD4+CD8+	% CM	% CCR7+	% annexin+
CAR-T	R16002	2.02 x 10^8^	73.5	1.49 x 10^8^	29.6/15.6/53.3	77.9	64.6	10.7
CAR-T	Rh2905	1.52 x 10^8^	77.8	1.21 x 10^8^	50.7/9.8/38.5	63.5	55.8	10.1
CAR-T	R14069	2.31 x 10^8^	73.8	1.71 x 10^8^	33.7/14/51.8	66.7	55.9	22.7
CAR-T	R16084	1.95 x 10^8^	66.4	1.27 x 10^8^	42/16.6/35.9	43.5	78.6	14.4
Depleted/CAR-T	R09072	2.06 x 10^8^	93.4	1.92 x 10^8^	21.7/6.13/72	81.7	68.4	13.2
Depleted/CAR-T	Rh3027	0.1 x 10^8^	93.1	0.093 x 10^8^	22.1/11.7/65.5	76.6	71.4	15.4
Depleted/CAR-T	Rh2997	0.42 x 10^8^	91.1	0.38 x 10^8^	37.5/12.6/49.5	85.4	75.7	28.0
Depleted/CAR-T	Rh2783	0.83 x 10^8^	88.3	0.73 x 10^8^	29.3/11.6/58.9	84.1	50.3	11.2

### Impact of anti-CD20 lymphodepletion

**Figure 2:**
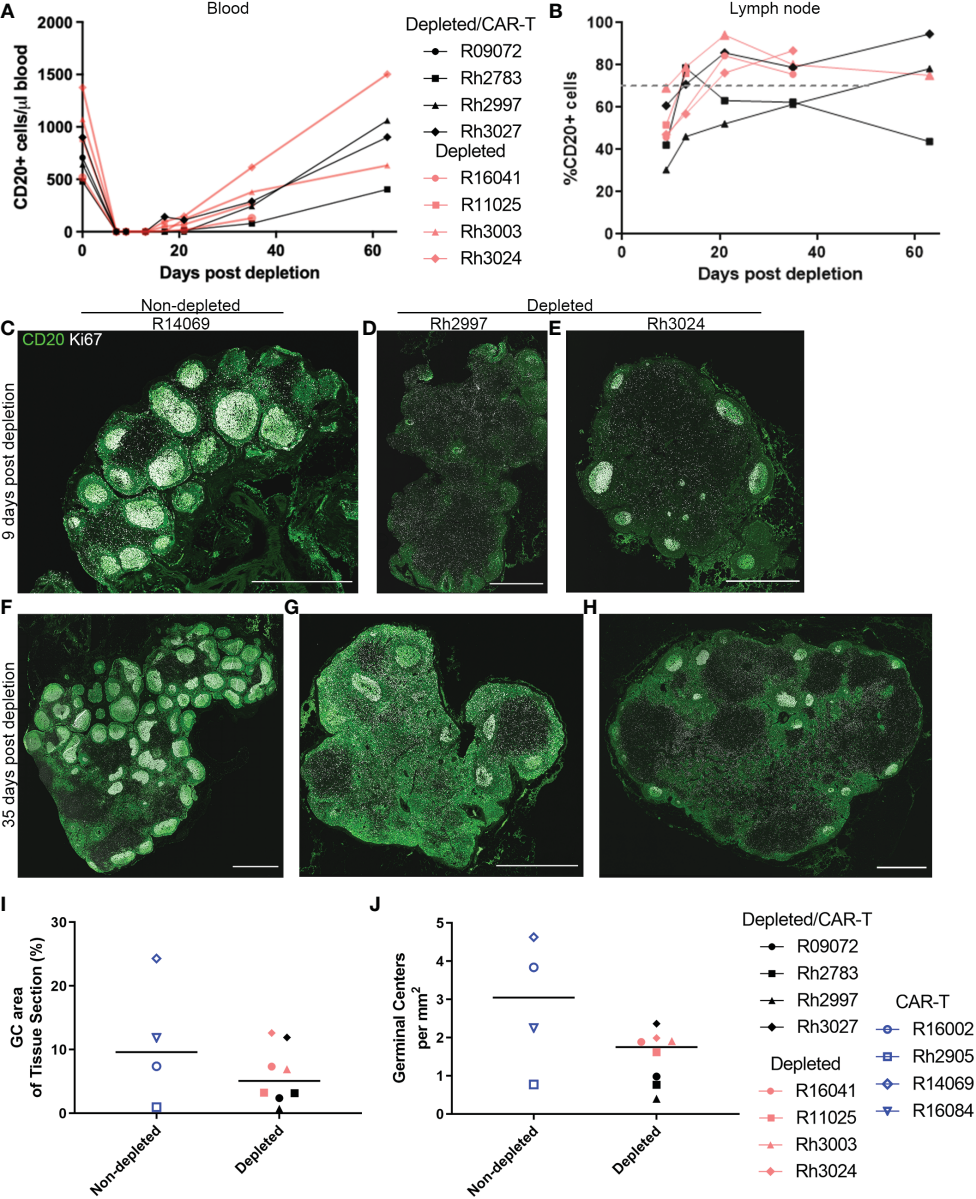
Anti-CD20 antibody treatment completely depleted CD20 in blood and partially depleted CD20 in lymph nodes. **(A, B)** Effect of anti-CD20 antibody treatment on CD20+ cells detected by flow cytometry in **(A)** PBMCs and **(B)** lymph node cells. For PBMCs, the cells were gated on live CD20+ before calculation of total CD20+ cells using lymphocyte number from CBC data. For lymph node, cells were gated on live, CD3-, CD20+ populations. The gray dashed line represents the mean percentage of CD20+ cells in non-depleted animals **(C–H)** Immunofluorescence images showing anti-CD20 staining (green) and anti-Ki67 (white) staining of lymph node sections corresponding to **(C–E)** 9 days post-depletion and **(F–H)** 35 days post-depletion for **(C, F)** CAR-T (non-depleted) animal R14069, **(D, G)** depleted/CAR-T animal Rh2997, and **(E, H)** depleted animal Rh3024. Scale bars are 1000 µm. **(I)** Effect of depletion on percent GC area of lymph node tissue sections. **(J)** Effect of depletion on number of GCs per tissue area.

We also examined whether a temporary depletion of CD20+ B cells would lead to increased expansion and persistence of CAR-T cells in rhesus macaques. Animals were treated with a low dose of anti-CD20 antibody (7 mg/kg). The anti-CD20 antibody was administered to both CAR-T treated (depleted/CAR-T) and control animals (depleted) 7 days prior to the infusion of the CAR-T cells. Effectiveness of the treatment was monitored in the blood and lymph node by evaluation of CD20+ cells by flow cytometry using both PBMCs and disaggregated lymph node cells. A representative flow plot showing CD20 detection is shown in **Supplementary Figure 1B**. Pre-depletion, the blood of study animals contained 480 to 1376 CD20+ cells/μl blood. After a single dose of anti-CD20 antibody, there was complete to near complete depletion of CD20+ cells in the blood detected at days 9 and 13 post-depletion, with a gradual recovery to pre-depletion levels by 2 months post-depletion (**Figure 2A**). When examining the cells from disaggregated lymph node biopsies, we did not have a pre-depletion biopsy for comparison; therefore, in order to determine the normal level of CD20+ cells, we examined CD20+ frequency in eight non-depleted animals. In non-depleted animals, there was an average of 70.3% CD20+ cells (range of 45 to 94%) in the CD3- population of the disaggregated lymph node cells. In CD20 depleted animals, a partial depletion of CD20+ cells was detected in disaggregated lymph nodes at day 9 post-depletion (**Figure 2B**). A gradual recovery of CD20+ cells was seen which reached normal levels (represented by the dashed gray line) by one month post-depletion (**Figure 2B**).

**Figure 3:**
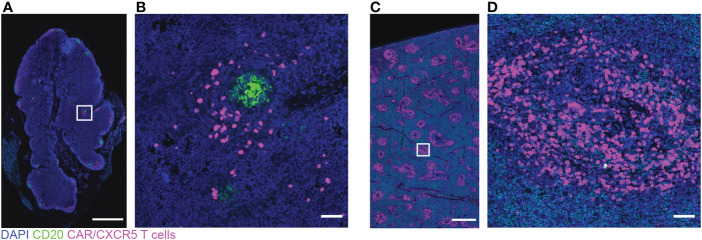
Low levels of CD20 and many CAR/CXCR5 T cells in depleted/CAR-T animal R09072 lymph node and spleen. RNAscope and immunofluorescence images showing CAR/CXCR5 RNA (magenta), anti-CD20 (green) and DAPI (blue) staining in lymph node **(A, B)** and spleen **(C, D)**. **(B, D)** are enlargements of the delineated areas in **(A, C)** respectively. Scale bars are 1000 µm for **(A, C)** and 50 µm for **(B, D)**.

To determine the effect of CD20 depletion on lymphoid follicles, we performed *in situ* immunofluorescence staining of lymph node sections with anti-CD20, to detect CD20+ B cells, and anti-Ki67, to detect proliferating B cells in GCs, which have been shown previously to be reduced following CD20 depletion (42). Nine days post-depletion, immunofluorescence staining of lymph nodes showed partial depletion of CD20+ cells and B cell follicles. **Figures 2C–H** shows immunofluorescence images of anti-CD20 and anti-Ki67 staining for tissue sections corresponding to 9 (**Figures 2C–E**) and 35 (**Figures 2F–H**) days post-depletion for non-depleted (**Figures 2C, F**) and depleted animals (**Figures 2D, E, G, H**). Interestingly, Rh2997 lymph node was most depleted by flow (**Figure 2B**), and immunofluorescence staining of lymph node sections at 9 days post-depletion showed reduced CD20 staining and only a few, small (<250 μm) clusters of GC Ki67+ cells in follicles (**Figure 2D**), which returned to normal levels by 35 days post-depletion (**Figure 2G**). In most depleted animals, lymph nodes at 9 days post-depletion showed some CD20 staining of B cells in follicles (**Figures 2D, E**) and clusters of GC Ki67+ cells (**Figure 2E**). To quantify depletion in situ, we determined the percentage of area of Ki67+ GC and the number of Ki67+ GCs at 9 days post-depletion in depleted animals compared to non-depleted animals. Overall, depleted animals had a lower median 5.08% (range 0.69-12.62%) GC area of lymph node tissue sections, compared to non-depleted animals, median 9.62% (range 0.96-24.32%) GC area of lymph node tissue sections (**Figure 2I**). We found the number of GCs in depleted animals to be a median 1.75 GCs/mm^2^ (range 0.40-2.36 GCs/mm^2^), and non-depleted animals had a median 3.05 GCs/mm^2^ (range 0.77-4.63 GCs/mm^2^) in lymph node sections (**Figure 2J**). We did not find a significant difference between non-depleted and depleted animals for percent GC areas of lymph node tissue sections (p=0.46) or number of GCs (p=0.15). Interestingly, *in situ* immunofluorescence staining with anti-CD20 using lymph node from the first treated animal (R09072), which was not available for flow cytometry, showed the greatest depletion of CD20+ cells and follicles *in situ* (**Figures 3A, B**). Notably, no CD20 staining was detected in the spleen of this animal (**Figures 3C, D**), indicating that CD20 depletion was greater in the spleen compared to lymph nodes.

To gain further insights into the composition of the follicles 9 days post-depletion, we stained selected lymph node sections with anti-CXCL13 and anti-CD79a (**Supplementary Figures 3A–F**). CXCL13 is the ligand for CXCR5 and is expressed by follicular dendritic cells (FDC). CD79a is an alternative marker to label B cells. CXCL13-stained FDC and CD79a+ B cells were detected in sections, confirming that, at 9 days post-anti-CD20 antibody treatment, there were detectable GC and B cells, that there was partial depletion of lymph node follicles (**Supplementary Figures 3B–D, F**), and that R09072 was the most depleted animal (**Supplementary Figure 3B**). CD20 depleted animal lymph nodes also showed bright CD20 staining not resembling B cells within follicles (**Figures 3A, B**; **Supplementary Figures 3G, H**) that was likely CD20 from recently killed B cells captured on FDC, as this has been described previously (42, 50). Anti-CD20 (**Supplementary Figure 3H**) appeared to brightly stain FDC, whereas anti-CD79a (**Supplementary Figure 3F**) did not stain FDC (compare to FDC staining, **Supplementary Figure 3E**). These results support earlier findings that CD20 is captured on FDC post-CD20 cell depletion.

### Impact of CD20 depletion on CAR-T immunotherapy

**Figure 4:**
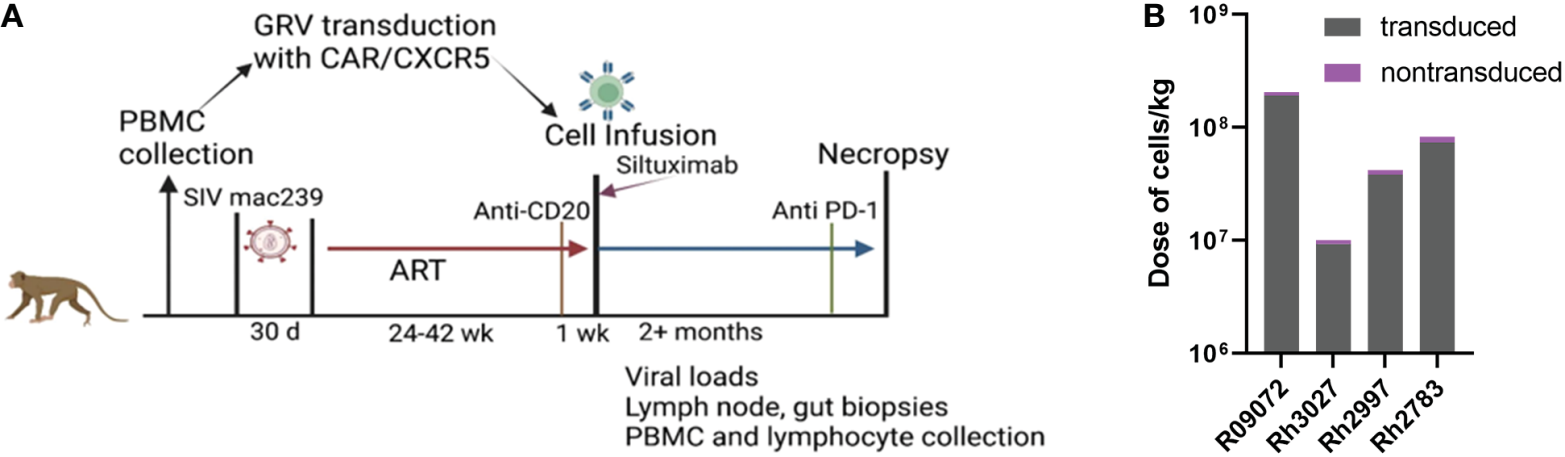
The CD20 depletion study. **(A)** The study design. **(B)** Each bar represents the total number of cells infused into each treatment animal with the number of transduced cells presented in gray and non-transduced cells presented in purple. **(A)** was created with BioRender.com.

The study design for the CD20 depletion/CAR-T study is presented in **Figure 4A**. It is similar to the previous CAR T-cell study with the exception that anti-CD20 antibody was administered a week prior to CAR-T infusion, Siltuximab was administered to the animals at the time of infusion, and anti-PD-1 was administered to some of the animals prior to necropsy (**Table 1**). As with the previous study, cells were collected from the animals prior to infection and CAR-T cells were produced using gammaretroviral transduction of autologous PBMCs. Cells highly co-expressed both the CAR and CXCR5 (median 92%; range 88-93%) and were primarily of a central memory phenotype (**Table 2**; **Supplementary Figure 1**). Cells were infused into the animals at the time of release from ART. Doses ranged from 0.1 to 2.0 x 10^8^ cells/kg for the depleted/CAR-T animals (**Table 2**; **Figure 4B**).

**Figure 5:**
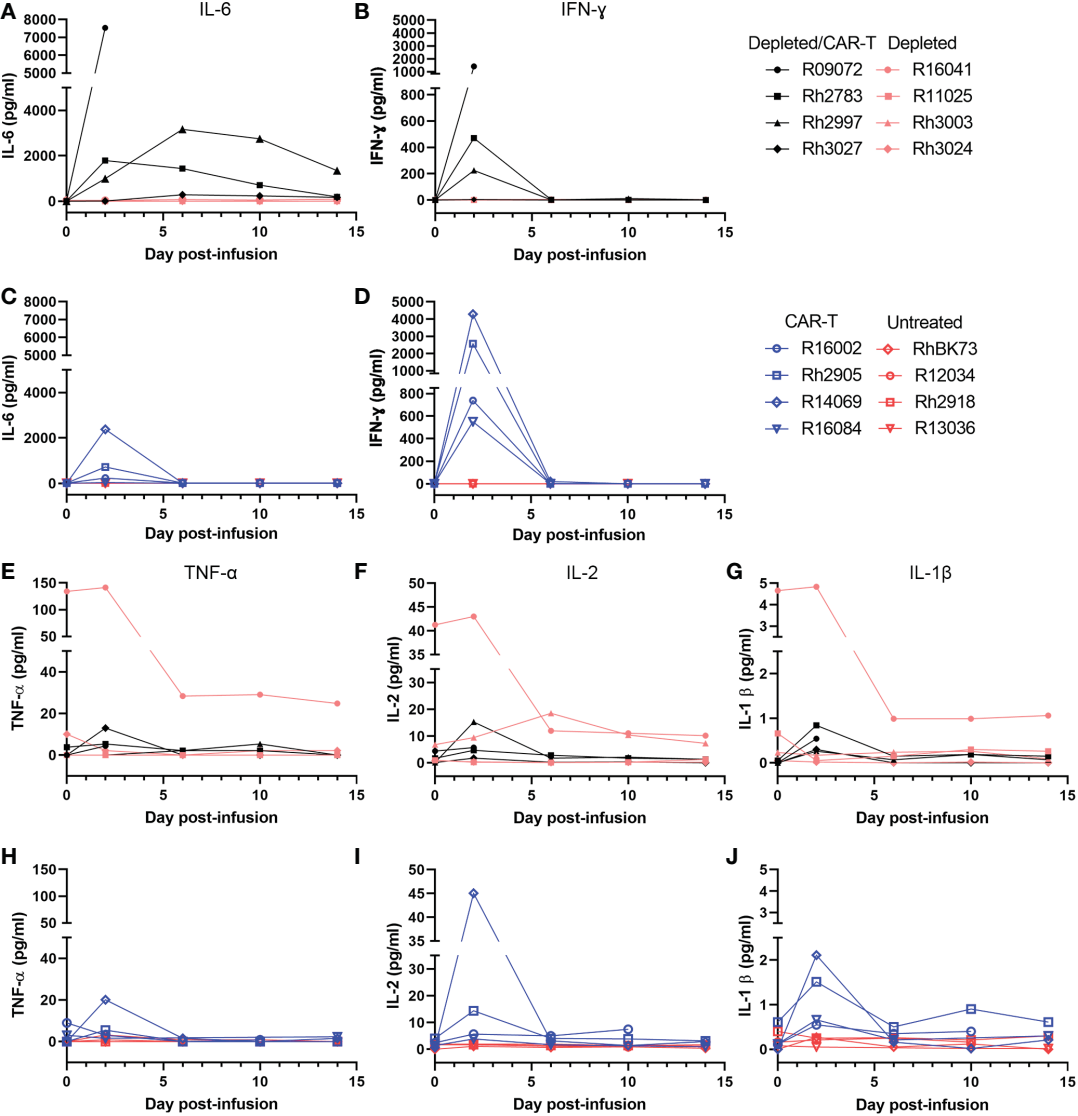
Level of cytokines in the serum of depleted and non-depleted animals as determined by a Luminex assay. All depleted/CAR-T animals are represented in black, depleted animals in peach, CAR-T in blue and untreated in red. **(A)** Level of IL-6 in the serum of the CD20-depleted animals over time. **(B)** Level of IFNγ in the serum of C20-depleted animals over time. **(C)** Level of IL-6 in the serum of non-depleted animals over time. **(D)** Level of IFNγ in the serum of non-depleted animals over time. **(E)** Level of TNFα in the serum of the CD20-depleted animals over time. **(F)** Level of IL-2 in the serum of the CD20-depleted animals over time. **(G)** Level of IL-1β in the serum of the CD20-depleted animals over time. **(H)** Level of TNFα in the serum of non-depleted animals over time. **(I)** IL-2 level in the serum of non-depleted animals over time. **(J)** IL-1β level in the serum of non-depleted animals over time.

Initially, the experimental plan was to use a high dose of ~2x10^8^ cells/kg CAR-T cells in both CAR-T and depleted/CAR-T treated animals to allow a direct comparison of the impact of anti-CD20 pre-treatment. However, the first depleted/CAR-T treated animal (R09072) developed cytokine release syndrome (CRS) requiring euthanasia at 2 days post-CAR-T infusion. Levels of IL-6 and interferon-ɣ were very high at the time of death (**Figures 5A, B**) while levels of TNF-ɑ, IL-2, and IL-1β all fell within the normal range for adult rhesus macaques (51) (**Figures 5E–G**). Necropsy results indicated damage to the liver and lungs. To help prevent any further severe CRS responses and assess the safety of the treatment, the remaining depleted/CAR-T animals received 11 mg/kg Siltuximab (anti-IL-6 antibody) at the time of cell infusion. In addition, the dose of infused cells was reduced twenty-fold for the next animal with a dose escalation for each subsequent animal (**Table 2**; **Figure 4B**). All animals had normal cytokine levels on the day of infusion. After CAR-T cell infusion, both CAR-T and depleted/CAR-T treated animals had elevated levels of IL-6 and interferon-ɣ (**Figures 5A–D**) while levels of IL-2, IL-1β, and TNF-ɑ all fell within the normal range for rhesus macaques (**Figures 5E–J**). For reference, in adult macaques, the normal range of IL-6 is 0-16.15 pg/ml; IFN-γ is 0-78.09 pg/ml; IL-2 is 1.78-234.1 pg/ml; IL-1β is 0-44.85 pg/ml and TNFα is 0-107.2 pg/ml (51) Despite treatment with Siltuximab, and lowered doses of CAR-T cells, the depleted/CAR-T cell treated animals showed high IL-6 levels comparable to the non-depleted CAR-T treated animals that did not receive Siltuximab (**Figures 5A, C**). The initial production of IL-6 was related to the dose of cells infused. While the CAR-T treated animals all showed an immediate drop back to normal levels of IL-6 by 6 DPT, the depleted/CAR-T treated animals showed a prolonged elevation of IL-6 (**Figure 5A**), suggesting a more severe cytokine response to the CAR-T cell infusion, possibly related to increased cell expansion.

### SIV levels in the CD20 depleted animals

**Figure 6:**
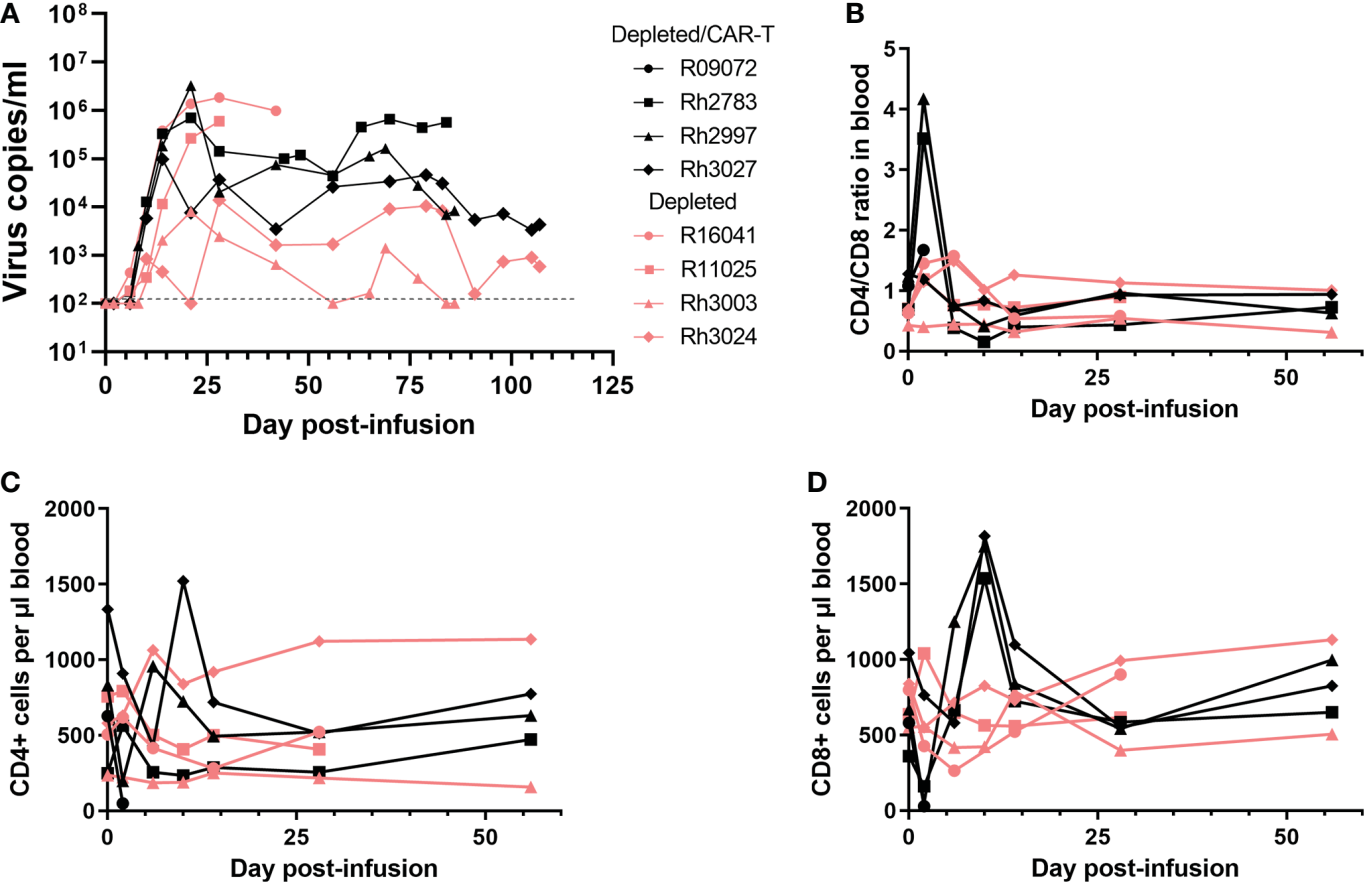
Impact of CAR-T cell infusion in CD20-depleted animals. **(A)** Virus copies per ml over time for each of the depleted/CAR-T (black) and depleted (peach) animals. Note R09072 is not shown on this graph because it was euthanized 2 days post-infusion. **(B)** CD4/CD8 ratios over time in the PBMCs, **(C)** CD4+ cell counts per μl blood and **(D)** CD8+ cell counts per μl blood. Each graph depicts the depleted/CAR-T animals in black and the depleted animals in peach.

**Figure 7:**
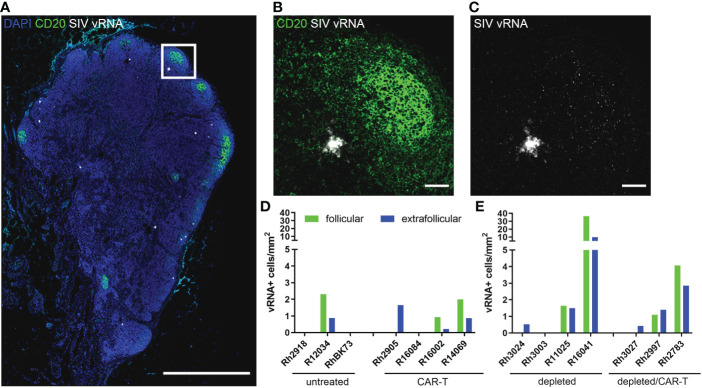
SIV vRNA+ cells detected at 28 DPT. **(A)** RNAscope and immunofluorescence image showing SIV vRNA (white), anti-CD20 (green) and DAPI (blue) staining in depleted animal R11025 lymph node, scale bar=1000 µm. **(B, C)** enlargement of delineated area in **(A)** showing anti-CD20 staining **(B)** and SIV vRNA **(B, C)**, including a vRNA+ cell and virions trapped on FDC, scale bars=50 µm. **(D, E)** Graphs showing follicular (green) and extrafollicular (blue) concentrations of SIV vRNA+ cells in non-depleted **(D)** and depleted **(E)** animal lymph nodes. Note R13036 28 DPT biopsy was not available.

The animals in the depletion study received doses of CAR-T cells that were 2-20 fold lower than our previous study (**Figure 4B**). Not surprisingly, we did not see a difference in viral loads between the depleted/CAR-T treated and depleted control animals (**Figure 6A**) or any long-term impact on CD4+ or CD8+ cell counts or CD4/CD8 ratios (**Figures 6B–D**). We observed a decrease in CD8+ cell counts and a corresponding increase in CD4+/CD8+ ratio at 2 days post-infusion and an increase in CD8+ cell counts at 10 days post-infusion in depleted/CAR-T animals (**Figures 6B, D**) and in CAR-T animals (not shown). There was no difference in follicular vRNA+ cell concentrations in lymph nodes in the depleted/CAR-T group compared to the control depleted animals (**Figures 7A–E**). Interestingly, the depleted/CAR-T and depleted control animals appeared to show overall higher vRNA+ cells in lymph nodes at 28 DPT (35 days post-depletion) than the untreated and CAR-T treated animals (**Figures 7D, E**). In order to assess the impact of partial CD20 depletion on SIV levels in macaques, we compared the viral loads of the depleted and non-depleted control animals (**Supplementary Figure 4A**). While variability is seen within each group, there is no significant difference between the viral loads of each group at 6 (p=0.31), 14 (p=0.88) or 28 (p=0.69) DPT (**Supplementary Figures 4B–D**). As a side note, two of the depleted control animals were lost prior to 50 DPT. One of the animals (R16041) had persistently high viral loads and was euthanized on day 45 by recommendation of the veterinarian. The other animal (R11025) died on day 42. Necropsy results suggest hypertrophic cardiomyopathy and pneumonia as primary causes of death.

### Detection of CAR-T cells in blood and tissues

**Figure 8:**
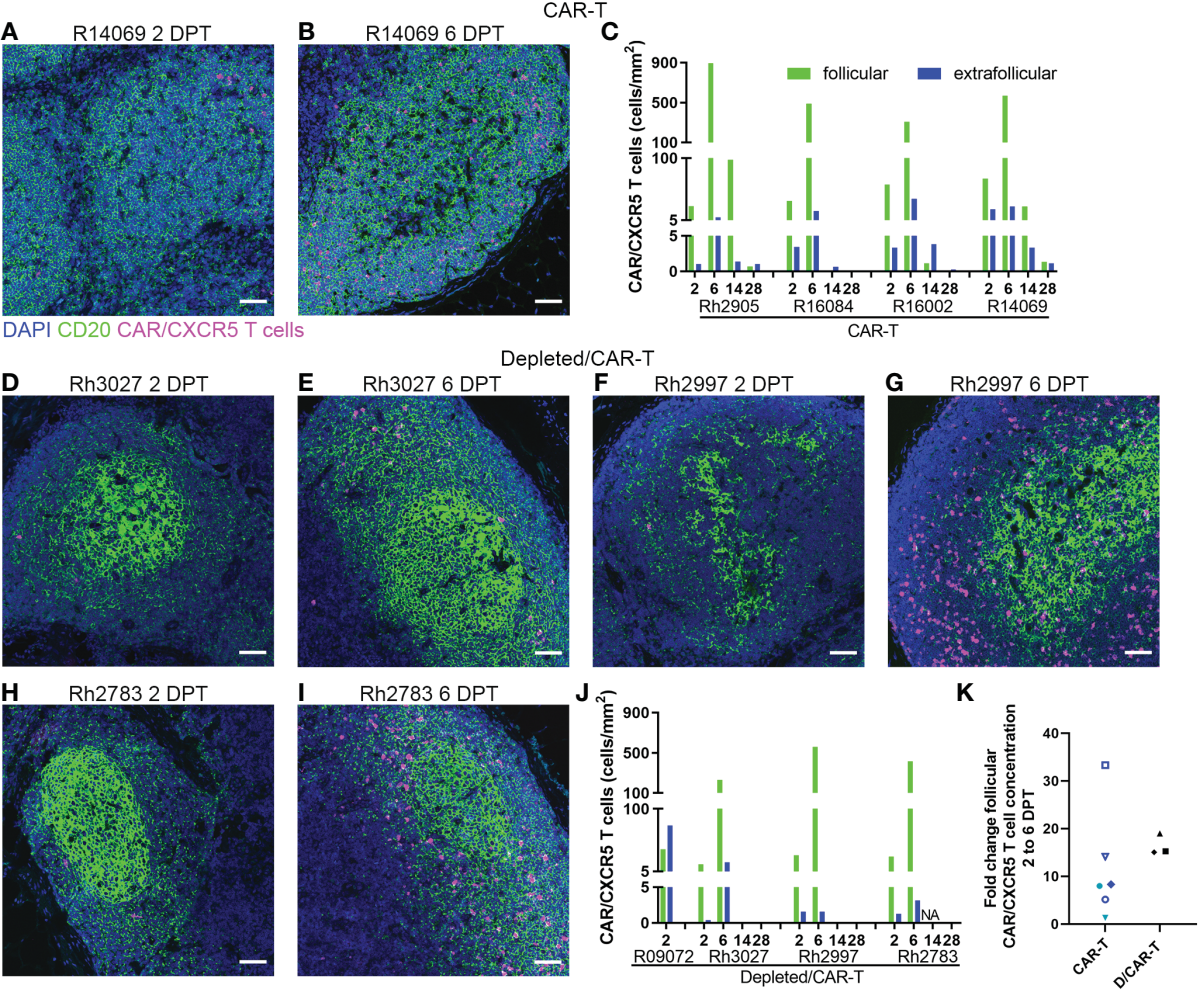
CAR/CXCR5 T cells localized to follicles in treated animals. **(A, B)** RNAscope and immunofluorescence images showing CAR/CXCR5 RNA (magenta), anti-CD20 (green) and DAPI (blue) staining in CAR-T animal R14069 at 2 **(A)** and 6 **(B)** DPT. Scale bars =50 µm. **(C)** CAR/CXCR5 T cell concentrations over time in follicular and extrafollicular regions of CAR-T animal lymph nodes determined by RNAscope. **(D–I)** RNAscope and immunofluorescence images showing CAR/CXCR5 RNA (magenta), anti-CD20 (green) and DAPI (blue) staining in depleted/CAR-T lymph node sections from 2 and 6 DPT. Scale bars =50 µm. **(J)** CAR/CXCR5 T cell concentrations over time in follicular and extrafollicular regions of depleted/CAR-T animal lymph nodes determined by RNAscope. NA indicates animal Rh2783 lymph node at 14 DPT was not available. **(K)** Fold change of follicular CAR/CXCR5 cell concentration from 2 to 6 DPT determined by RNAscope for CAR-T (including two earlier pilot animals) and depleted/CAR-T animals.

**Figure 9:**
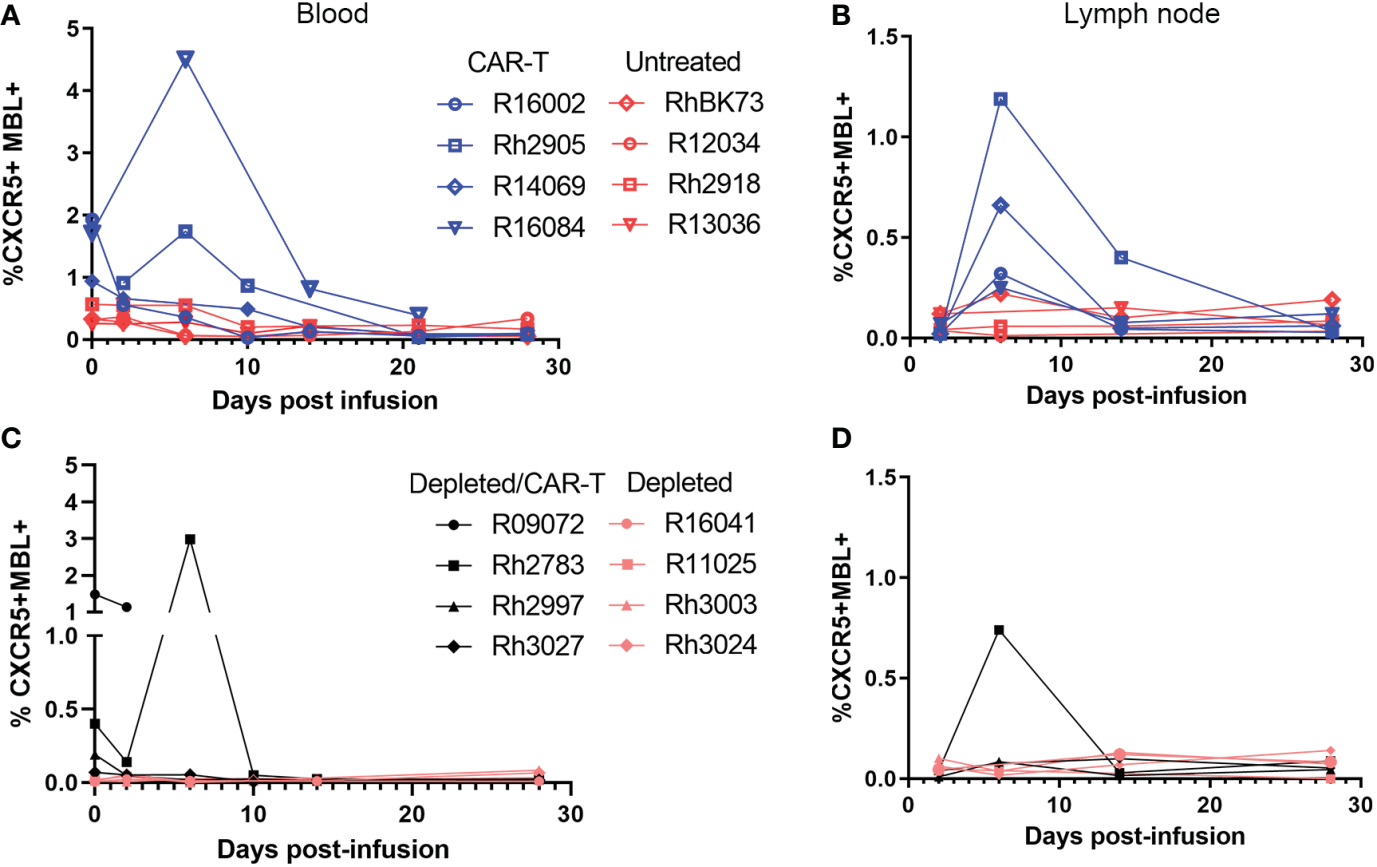
Levels of CAR/CXCR5 T cells post-infusion as determined by flow cytometry. Levels of CAR/CXCR5 T cells in **(A)** PBMCs and **(B)** disaggregated lymph node cells in non-depleted animals. CAR-T treated animals are represented in blue and untreated animals are represented in red. **(C, D)** Levels of CAR/CXCR5 T cells in **(C)** PBMCs and **(D)** disaggregated lymph node cells in CD20 depleted animals. Depleted/CAR-T treated animals are represented in black and depleted/untreated animals are represented in peach.

In the CAR-T group, CAR-T cells accumulated in follicles and were detected *in situ* in lymph node biopsies to 14 (n=3) and 28 DPT (n=2) (**Figures 8A–C**). Blood and disaggregated lymph nodes examined by flow cytometry, showed similar findings (**Figures 9A, B**). Similar levels of CAR-T cells were detected in lymph node sections *in situ* in both CAR-T (**Figures 8A–C**) and depleted/CAR-T groups (**Figures 8D–J**) despite the depleted/CAR-T group receiving substantially lower doses of cells (**Table 2**; **Figures 1C**, **4B**). In the depleted/CAR-T group, CAR-T cells accumulated in and around residual/new follicles (**Figures 8D–I**, **3A**), and, in contrast to the CAR-T group (**Figure 8C**), were only detected *in situ* through 6 DPT (n=3) (**Figure 8J**). In spleen sections from the high dose animal sacrificed 2 days post-treatment (R09072), abundant CAR T cells were detected in the white pulp (**Figures 3C, D**). There were substantially higher concentrations of CAR-T cells in the spleen compared to lymph node sections (**Figure 3**), consistent with our earlier findings (22). In PBMCs isolated from the depleted/CAR-T group and examined by flow cytometry, CAR-T cells were detectable only in the first week and only in the animals that received the highest doses of CAR-T cells (Rh2783 and R09072, **Figure 9C**). The phenotype for the CAR-T cells in the blood in depleted/CAR-T treated animal Rh2783 changed markedly after infusion. The cells dropped from 91.3% to 36.4% central memory phenotype between 0 DPT and 6 DPT. For comparison, the day 6 central memory population ranged from 47.9-60.3% in the non-depleted CAR-T treated animals. Similar levels of CAR-T cell detection were seen with disaggregated lymph node cells for Rh2783 (**Figure 9D**). Note that no fresh lymph nodes were collected for disaggregation at 2DPT for the animal that received the highest dose of CAR-T cells, R09072. In flow cytometry assays, both the level and the persistence of the CAR-T cells were lower in the depleted CAR-T animals than in the non-depleted CAR-T animals (**Figures 9C, D** compared to **Figures 9A, B**). In contrast to the blood, the central memory phenotype was maintained in CAR-T cells found in disaggregated lymph node cells from Rh2783; we found that 70.3% of the CAR-T cells from 6 DPT had a central memory phenotype. Similar findings were detected in the 6 DPT lymph node samples from the non-depleted study animals, which showed a range of 58-73% of the CAR-T cells with a central memory phenotype. By RNAscope, we examined the fold change of CAR-T cells in follicles from 2 to 6 DPT. We combined data from CAR-T cell treated animals in this study, with data from our previous CAR-T cell treatment study and found that CAR-T cells increased in follicles a median of 15.2-fold in depleted/CAR-T treated animals compared to 8.1-fold in CAR-T treated animals (**Figure 8K**). Although we did not have sufficient numbers of depleted/CAR T cell treated animals to expect to find significance using a nonparametric Mann Whitney test; the descriptive statistics from the small cohort suggest that CD20 depletion may impact the rate of expansion and/or migration of CAR-T cells into follicles.

We administered anti-PD-1 antibodies to the depleted CAR-T animals at a late timepoint (**Figure 4A**; **Table 1**) to assess whether any reversal of T- or CAR-T cell exhaustion might be observed. Examination of viral loads at the time of administration and 4 days later, show no impact on SIV viral loads. Additionally, by flow cytometry, no CAR-T cells were detectable in the PBMCs 4 days after anti-PD-1 antibody administration.

### Expansion of the CAR-T cells

**Figure 10:**
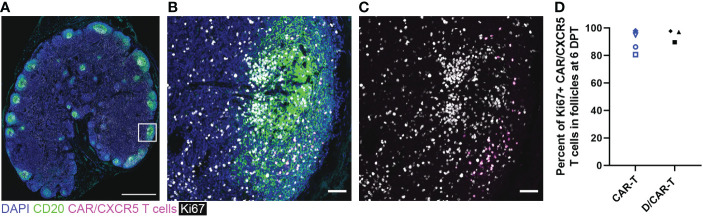
CAR/CXCR5 T cells were proliferating at 6 DPT. **(A)** RNAscope and immunofluorescence image showing CAR/CXCR5 RNA (magenta), anti-CD20 (green), anti-Ki67 (white) and DAPI (blue) staining in depleted/CAR-T animal Rh3027 lymph node. Scale bar=1000 µm. **(B, C)** Enlargement of the delineated area in **(A)** showing costaining of CAR/CXCR5 T cells (magenta) with anti-Ki67 (white). Scale bars = 50 µm. **(D)** Percent of Ki67+ CAR/CXCR5 T cells at 6 DPT in depleted/CAR-T and CAR-T animal lymph node follicles.

We evaluated whether CAR-T cells expressed the activation and cell division marker Ki67 using both flow cytometry and *in situ* using RNAscope combined with immunofluorescent staining. Though CAR-T cells were detected in the lymph node of only one depleted animal by flow cytometry (Rh2783) at 6 DPT, we found that the majority were Ki67+ (79%). By IHC, almost all CAR-T cells were Ki67+ at 6 DPT in both the CAR-T and depleted/CAR-T groups (**Figure 10**). **Figures 10A–C** show representative images of CAR-T cells with CAR/CXCR5 RNA and Ki67 co-staining, which is quantified in **Figure 10D**. CAR-T treated animals had a median 91% Ki67+ CAR-T cells and depleted/CAR-T treated animals had a median 97% Ki67+ CAR-T cells. The CAR-T cells being Ki67+ indicates *in situ* proliferation, and in combination with the increased abundance of cells from days 2 to 6 post-treatment, suggests that the CAR-T cells underwent expansion.

### Detection of anti-CAR antibodies in the serum of treated animals

**Figure 11:**
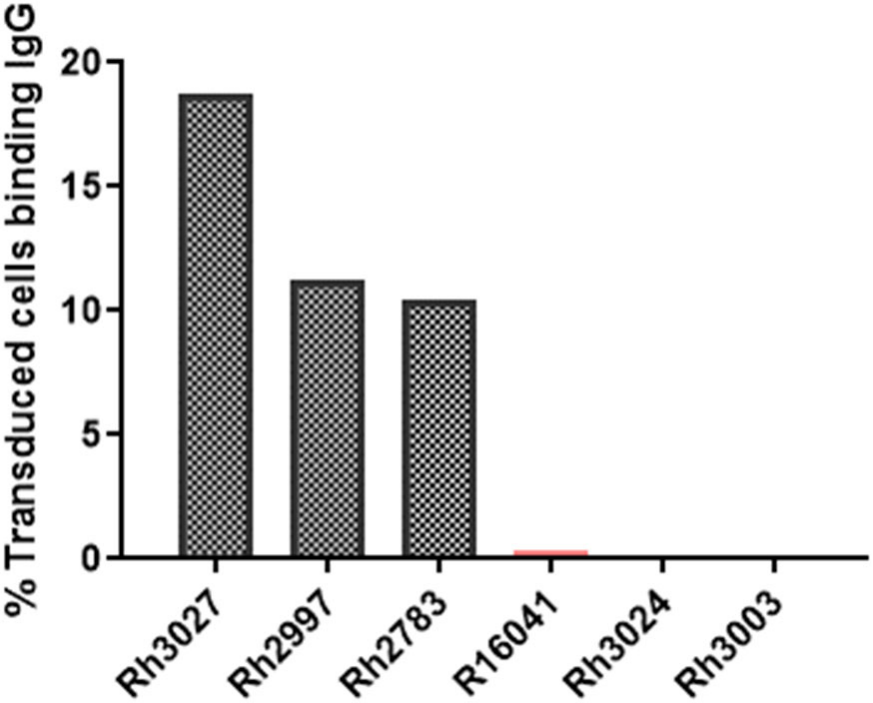
Presence of anti-CAR antibodies in the serum of CD20-depleted, CAR-T cell treated animals at 56 DPT. Anti-CAR IgG was identified by incubation of heat-inactivated 56 DPT serum with cells expressing the CD4MBL CAR followed by flow cytometry to measure anti-IgG binding to CD4+MBL+ cells. Anti-IgG was measured in the serum of depleted/CAR-T treated (black) and depleted (peach) animals.

The production of anti-CAR antibodies has a potential impact on the persistence of immunotherapeutic cells. Anti-CAR IgG antibodies were detected in the serum of CAR/CXCR5-treated animals, including the non-depleted animals presented in this study, as outlined in a previous manuscript (49). In the CD20-depleted animals, at 56 DPT, all three of the CAR/CXCR5-treated animals also had detectable levels of anti-CAR IgG antibodies in their serum (**Figure 11**). No antibodies were detected in the serum of the control animals.

## Discussion

In this study, macaques were treated with CD4-MBL CAR/CXCR5 T cells with or without an anti-CD20 antibody pre-treatment. Findings from the non-CD20-depleted CAR-T treated animals, along with our earlier studies, indicate that administration of CAR/CXCR5 T cells can be safe and is potentially efficacious in promoting sustained remission of SIV infection. Future mechanistic studies will help to better understand responding and nonresponding CAR-T cell treated animals.

We hypothesized that temporary depletion of B cells would provide space for immunotherapeutic cells to expand and promote engraftment. While the CD4-MBL-CAR/CXCR5 T cells that we infused appear to have gone through a proliferative burst from days 2-6, they all died shortly thereafter, and there was no impact on viral loads. As described below, the loss of CAR T cells may have been intrinsic to the CAR T cells used. Nonetheless, anti-CD20 antibody treatment may allow CAR-T cells to rapidly expand to a higher fold than in non-depleted animals. Data supporting this include observation of higher median fold increases of follicular CAR-T cells in the depleted/CAR-T cell treated animals compared to the non-depleted CAR-T cell treated animals. Second, CAR-T cell infusion in depleted/CAR-T animals led to a prolonged induction of high levels of IL-6 and IFN-γ that was not detected in the non-depleted CAR-T cell treated animals. High levels of IL-6 and IFN-γ are commonly associated with the onset of CRS and expansion of CAR-T cells (52, 53). Finally, the CAR-T cells in the depleted/CAR-T cell treated animals, but not non-depleted CAR-T cell treated animals, promptly died after the dramatic rise in numbers from day 2 to 6. CD8 T cells that undergo increased proliferative bursts/rounds of cell division are associated with decreased cell fitness and loss of cells (54, 55). Taken together, these data support anti-CD20 antibody pre-treatment leading to increased expansion of CAR-T cells.

In CD20 depleted animals, complete depletion was observed in the blood and partial depletion was observed in the lymph nodes nine days after administration of 7 mg/kg anti-CD20. Notably, we used the L26 antibody for immunofluorescence, which detects an intracellular epitope of CD20 that should not be blocked by therapeutic anti-CD20 (56). Previous studies have used varying amounts of anti-CD20 to deplete B cells in blood and lymph nodes. Infusion of 1.6 or 6.4 mg/kg rituximab (human anti-CD20 antibody) in cynomolgus macaques resulted in over 95% depletion of B cells in peripheral blood for 8 days and variable CD20 depletion in lymph nodes, resulting in 34-77% depletion of lymph node B cells seen 2 and 4 weeks after treatment (57). Typically blood is readily depleted, but it can be difficult to completely deplete lymph nodes especially with low doses, because of limited delivery of antibodies into the lymph nodes (45). Three weekly doses of 20 mg/kg rituximab were shown to significantly deplete B cells in the peripheral blood and lymph nodes of rhesus macaques, also resulting in decreased Ki67+ B cells in GCs (42), and 50 mg/kg doses of rituximab depleted CD20+ cells in blood and lymph node of African green monkeys (44). Our observations of B cell depletion in blood and partial depletion in lymph nodes are consistent with administration of a lower dose of anti-CD20. Interestingly, the lymph node of CD20 depleted/CAR-T treated animal R09072 appeared most depleted by anti-CD20 antibody immunofluorescence and no CD20 staining was observed in the spleen of that animal, suggesting the spleen could be more depleted than the lymph node.

In CD20 depleted animals at 9 days post-depletion, in addition to CD20 and CD79a staining of B cells, we observed bright follicular CD20 staining (but not CD79a staining) that did not resemble B cell morphology, but instead FDC morphology, and corresponded to areas that stained brightly with anti-CXCL13 antibodies. Previous studies have shown that after CD20 depletion, CD20 is captured on FDC (42, 50), which accounts for the bright CD20 staining with FDC morphology and association with CXCL13 staining (58) that we observe in follicles of depleted animals.

Previous studies showed the impact of B cell depletion on the development of antiviral antibody responses. Miller et al. (59) depleted B cells by administering 50 mg/kg of rituximab every three weeks and infected macaques with SIVmac239. In this study, some monkeys did not develop anti-SIV antibody responses and rapidly progressed to AIDS, whereas some monkeys did develop anti-SIV antibodies and maintained moderate plasma viral loads (59). Schmitz et al. administered three weekly doses of 20 mg/kg rituximab, infected macaques with SIVmac251, and found delayed development of SIV neutralizing antibodies (42). Gaufin et al. depleted B cells by administering 50 mg/kg of rituximab every three weeks for 2-5 months and infected macaques with SIVssmD215. They found, in macaques with incomplete CD20 tissue depletion, antibody titers were not different from non-depleted controls and that there were no differences in viral load between depleted and non-depleted animals (60). In contrast to these studies, we administered a much lower dose of anti-CD20 antibody, and it was administered just before cessation of ART, instead of before SIV infection. Notably, anti-CD20 antibodies like rituximab do not target CD20- plasma cells, so long-lived plasma cells could contribute to maintenance of existing antibody responses after B cell depletion (61). While we did not measure anti-SIV antibodies in our treatment animals, we found no significant difference in viral loads post ATI when comparing the non-depleted and depleted control animals. This finding suggests that the temporary depletion in the blood and the partial and temporary depletion in the lymph node did not have an effect on the ability of the animals to respond to SIV infection. Additionally, as with our previous study (49), we found that the animals in the CD20 depletion study had anti-CAR IgG antibodies in their serum at 56 DPT. This finding suggests that the ability of the animals to develop antibodies to the CAR was not impeded by anti-CD20 treatment, perhaps due to the limited depletion of the lymphoid follicles.

As mentioned above, the CAR-T cells in the depleted/CAR-T treated animals became undetectable shortly after rapidly increasing in number the first week post infusion, and this proliferative burst may have led to decreased cell fitness and death. We also considered other mechanisms that may have contributed to the loss of CAR-T cells. The CAR-T cells in this CD20 depletion study were 11-28% annexin+ indicating that some were in the process of undergoing apoptosis. However, those percentages do not differ greatly from the percentages seen in the non-depleted CAR-T cell treated animals (10-23% annexin+), so increased annexin+ levels in the infused cell products does not seem a likely explanation of the quicker loss of cells in the depletion study. Since the animals developed anti-CAR antibodies, which likely reacted with the D1/D2 domains of CD4 on the CAR as in our previous study (49), it is possible that these antibodies impacted the long-term viability of the CAR-T cells and may also have impacted the ability of the CAR-T cells to bind to SIV-infected cells thereby decreasing their efficacy. However, the loss of the cells after 6 DPT, which precedes the development of an IgG response and detectable viral recrudescence, suggests that mechanisms other than antibody mediated cell killing are primarily responsible for the failure to persist. Exhaustion of CAR-T cells commonly occurs due to excessive exposure to antigen or to tonic signaling in the absence of antigen (62). In this study, the PBMCs used to produce the CAR-T cells were collected prior to infection, so they were unlikely to be exhausted. Excessive antigen exposure post-infusion is also an unlikely explanation for the cell loss in these studies since the cells are lost prior to the detection of viral recrudescence. However, the infused CAR-T cells may have had limited persistence because they failed to be stimulated by their antigen due to the timing of infusion on the day of release from ART. Unlike the CD19 CAR-T cell treatment of lymphoma and leukemia, antigen is scarce in SIV-infected animals shortly after release from ART. While the cells may retain some initial proliferative capacity, they may fail to be antigenically stimulated at an early timepoint and may not persist. Persistence may be improved by infusing the CAR-T cells a few days after ART release to allow SIV infected cells to recrudesce and stimulate the CAR-T cells. Another strategy to increase persistence of CAR-T cells, proposed by Rust et al. (63), could be to co-infuse SIV antigen presenting cells along with the CAR-T cells. Another possible explanation for the rapid early cell death of the CAR-T cells in the depleted/CAR-T animals is the functional phenotype arising from the CD28 costimulatory signaling domain used in the CAR. CAR-T cells with a CD28 costimulatory domain have increased effector function with increased production of cytokines, but have increased use of glycolytic pathways and are more likely to become exhausted and be eliminated (64–66). By contrast, CAR-T cells with a 4-1BB signaling domain have characteristics of central memory cells utilizing oxidative metabolism with a propensity to divide more slowly and persist longer (64, 65, 67). It is possible that CAR-T cells with 4-1BB signaling domains might expand more slowly and persist longer after CD20 depletion.

CAR-T cells can induce cytokine release syndrome (CRS) with symptoms that include fever, hypotension, tachycardia, hematologic cytopenias and, eventually, organ damage. The damaging effects of CRS are due to the release of cytokines by the infused CAR-T cells interacting with their target cells or other immune cells, such as monocytes, macrophages, NK cells, T cells, and dendritic cells, responding to the CAR-T cells (68–70). High levels of IL-6 are commonly associated with severity of CRS (52). CRS onset usually coincides with peak CAR-T cell expansion and occurs earlier in subjects treated with a CD28 CAR than a 4-1BB CAR (53). In clinical trials, most successful CAR-T cell infusions, with expansion and persistence of the CAR-T cells, led to induction of CRS (71). CRS is effectively reversed with IL-6 blockade, and the best studied treatment is the IL-6 receptor antagonist (Tocilizumab) (11, 12) IL-6 blockade with Tocilizumab does not appear to impact the effectiveness or persistence of CAR-T cells (69). In leukemia patients receiving CD19 CAR-T cells, engraftment or persistence of CD19 CAR-T cells was not impacted by use of Tocilizumab for CRS (72). In a study of lymphoma patients receiving CD19 CAR-T cells, Tocilizumab did not impact CAR-T cell persistence (73). Corticosteroids can also be used to reduce CRS toxicity if improvement is not observed with IL-6 receptor antagonist, but they may inhibit CAR-T cell persistence and are not a treatment of choice (70). In our previous study (22) and the non-depleted animals in this study, we observed increases in IL-6 post-infusion, but the levels peaked at 2 days and decreased back to normal at 6 days after cell infusion. No clinical signs of CRS were noted, and the use of IL-6 blockade was unnecessary. The first depleted/CAR-T animal was euthanized because of an apparent CRS response with extremely high levels of IL-6. The remaining depleted/CAR-T animals were treated with the anti-IL-6 antibody Siltuximab on the day of infusion. We used Siltuximab as a CRS prevention strategy in these studies due to the limited availability of Tocilizumab. While we expect that Siltuximab would similarly not impact CAR-T cell persistence, we acknowledge that an impact is possible. These animals had very high levels of IL-6 which were elevated for a prolonged period, despite the treatment with Siltuximab on the day of infusion. Although no clinical signs of CRS were noted in these animals, the prolonged, elevated levels of IL-6 suggest that the cells may have been expanding, perhaps due to additional space created by CD20 depletion, even though they failed to persist.

While these studies indicate that CD20+ B cell depletion may impact trafficking and expansion of CAR-T cells, we did not find that CD20 depletion enhanced persistence of the CD4-MBL/CAR-T cells which contain a CD28 signaling domain. Future CD20 depletion studies are needed to evaluate the expansion and persistence of other CAR-T cells bearing alternative signaling modalities. Future studies should incorporate an enhanced phenotype analysis of the infused cells in order to better understand mechanisms that lead to persistence or the failure to persist. Refinement of the timing of CAR-T cell infusion may be necessary for development of a successful therapy. For safety, given the high IL-6 levels induced by CAR T cells infused into CD20 depleted animals, and potential CRS development, future studies should incorporate potential CRS inhibitory drugs as are commonly used in clinical CAR-T cell trials.

## Data availability statement

The raw data supporting the conclusions of this article will be made available by the authors, without undue reservation.

## Ethics statement

The animal study was reviewed and approved by University of Wisconsin-Madison IACUC committee.

## Author contributions

MP and ES (equal): writing original draft, investigation, data curation, supervision, formal analysis, methodology, visualization; ZQ and BD: investigation, formal analysis, writing review and editing; JB and IG-B: investigation and data curation; HA: Investigation, data curation, formal analysis; ER: resources, funding acquisition, project administration; AR: formal analysis, writing review and editing; PS: Conceptualization, funding acquisition, writing original draft, supervision, project administration, resources. All authors contributed to the article and approved the submitted version.

## Supplementary material

Supplementary Figure 1 Flow cytometry gating strategies. **(A)** Gating strategy for day of infusion CAR-T cells (MBL+ CXCR5+), central memory cells (CD95+CD28+), CCR7+ cells. Cells from Rh2783 are shown **(B)** Representative flow plot for CD20+ cells in PBMCs. Rh2997 is shown at 9 and 35 days post-depletion. **(C)** Gating strategy for post-infusion detection of CAR-T cells. Day of infusion (D0) and 6 DPT (D6) are shown for Rh2783

Supplementary Figure 2 Viral loads of non depleted animals that were untreated or CAR-T treated at 3, 4 or 5 months post-infusion. Each point represents one animal. Untreated animals are from the current study (red) or a previous study (maroon) or from the current CAR-T treated group (blue) or a previous CAR-T study (maroon). The dashed line is at 1000 virus copies/ml for reference.

Supplementary Figure 3 B cells and CXCL-13 producing cells detected in lymph node sections with anti-CD79a (cyan) and anti-CXCL13 (yellow) staining at 9 days post-depletion in **(A)** CAR-T (non-depleted) animal R14069, **(B)** depleted/CAR-T animal R09072, **(C)** depleted animal Rh3024, and **(D)** depleted/CAR-T animal Rh2997. **(E, F)** are enlargements of the delineated area in **(D)** showing anti-CXCL13 **(E)** and anti-CD79a staining **(F)**. The arrow in **(E)** indicates FDC stained with anti-CXCL13. **(G)** shows anti-CD20 staining in a section near the section from **(D)** and **(H)** is an enlargement of the delineated area in **(G)**. The arrow in **(H)** indicates CD20 captured on FDC. Scale bars are 1000 µm for **(A–D, G)** and 50 µm for **(E, F, H)**

Supplementary Figure 4 Comparison of viral loads over time. **(A)** Viral loads are presented over time after ART interruption in the non depleted (light gray) and depleted (peach) control animals. Comparison of viral loads at **(B)** 6 days, **(C)** 14 days and **(D)** 28 days post ART interruption. The bars represent the median values for each data set.
