# Supplementary figures and images for: Assessment of anti-CD20 antibody pre-treatment for augmentation of CAR-T cell therapy in SIV-infected rhesus macaques
Source: Front Immunol. 2023 Feb 7;14:1101446. doi: 10.3389/fimmu.2023.1101446 (PMC9941136; doi:10.3389/fimmu.2023.1101446)

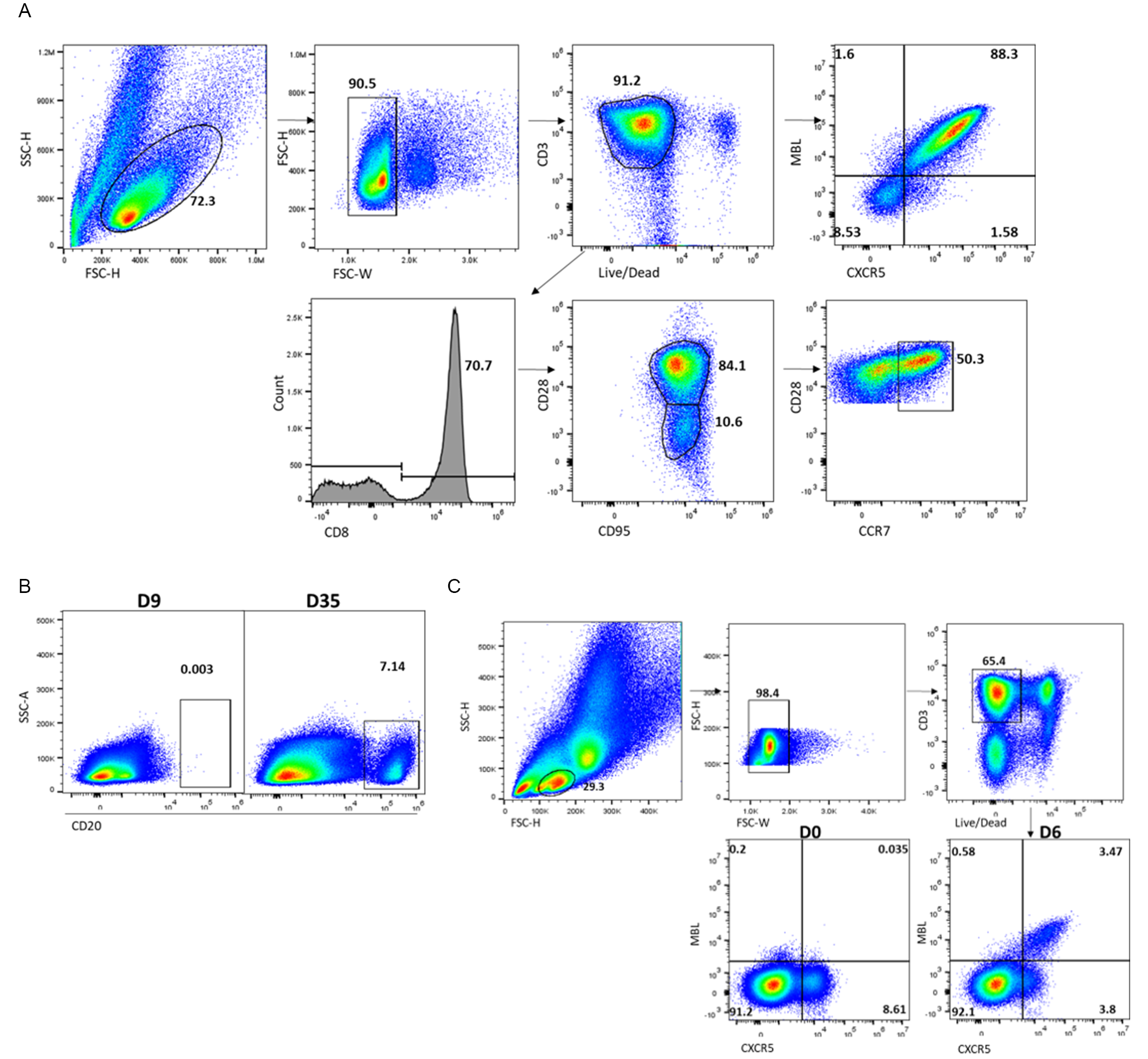

Supplement: Supplementary Figure 1 — Flow cytometry gating strategies. (A) Gating strategy for day of infusion CAR-T cells (MBL+ CXCR5+), central memory cells (CD95+CD28+), CCR7+ cells. Cells from Rh2783 are shown (B) Representative flow plot for CD20+ cells in PBMCs. Rh2997 is shown at 9 and 35 days post-depletion. (C) Gating strategy for post-infusion detection of CAR-T cells. Day of infusion (D0) and 6 DPT (D6) are shown for Rh2783 [file Image_1.tif]

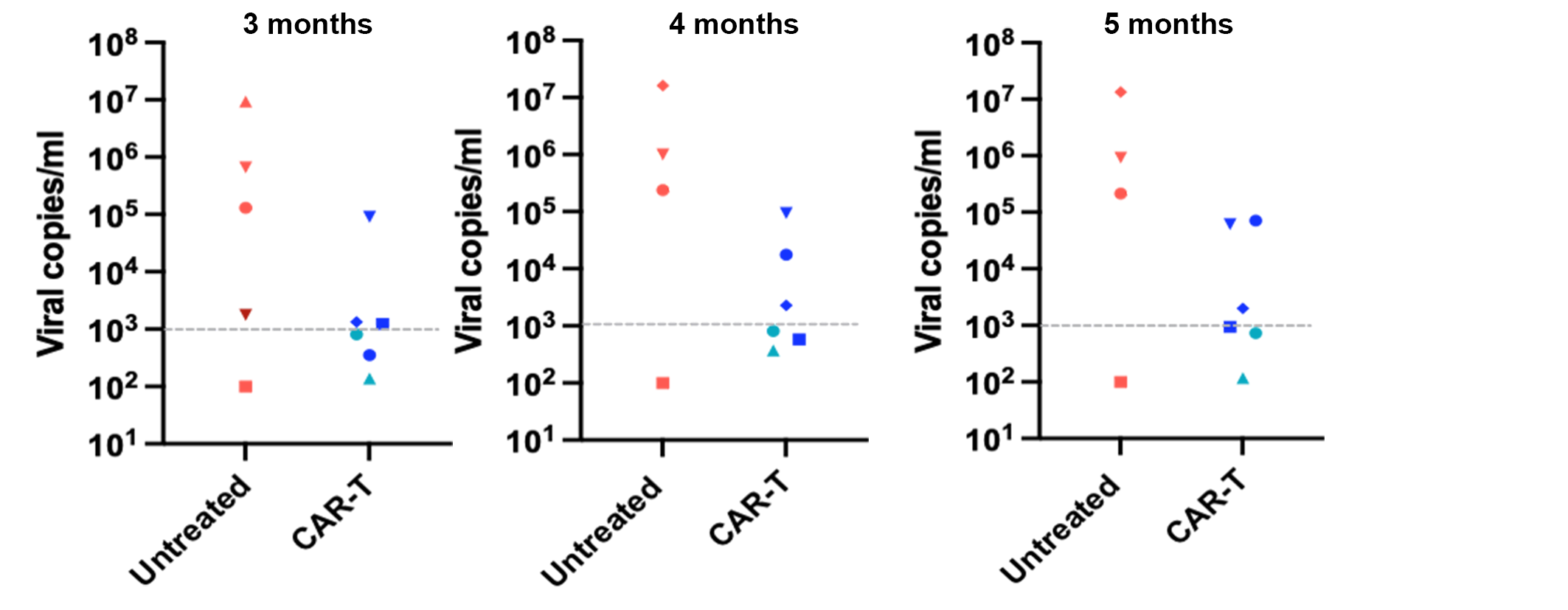

Supplement: Supplementary Figure 2 — Viral loads of non depleted animals that were untreated or CAR-T treated at 3, 4 or 5 months post-infusion. Each point represents one animal. Untreated animals are from the current study (red) or a previous study (maroon) or from the current CAR-T treated group (blue) or a previous CAR-T study (maroon). The dashed line is at 1000 virus copies/ml for reference. [file Image_2.tif]

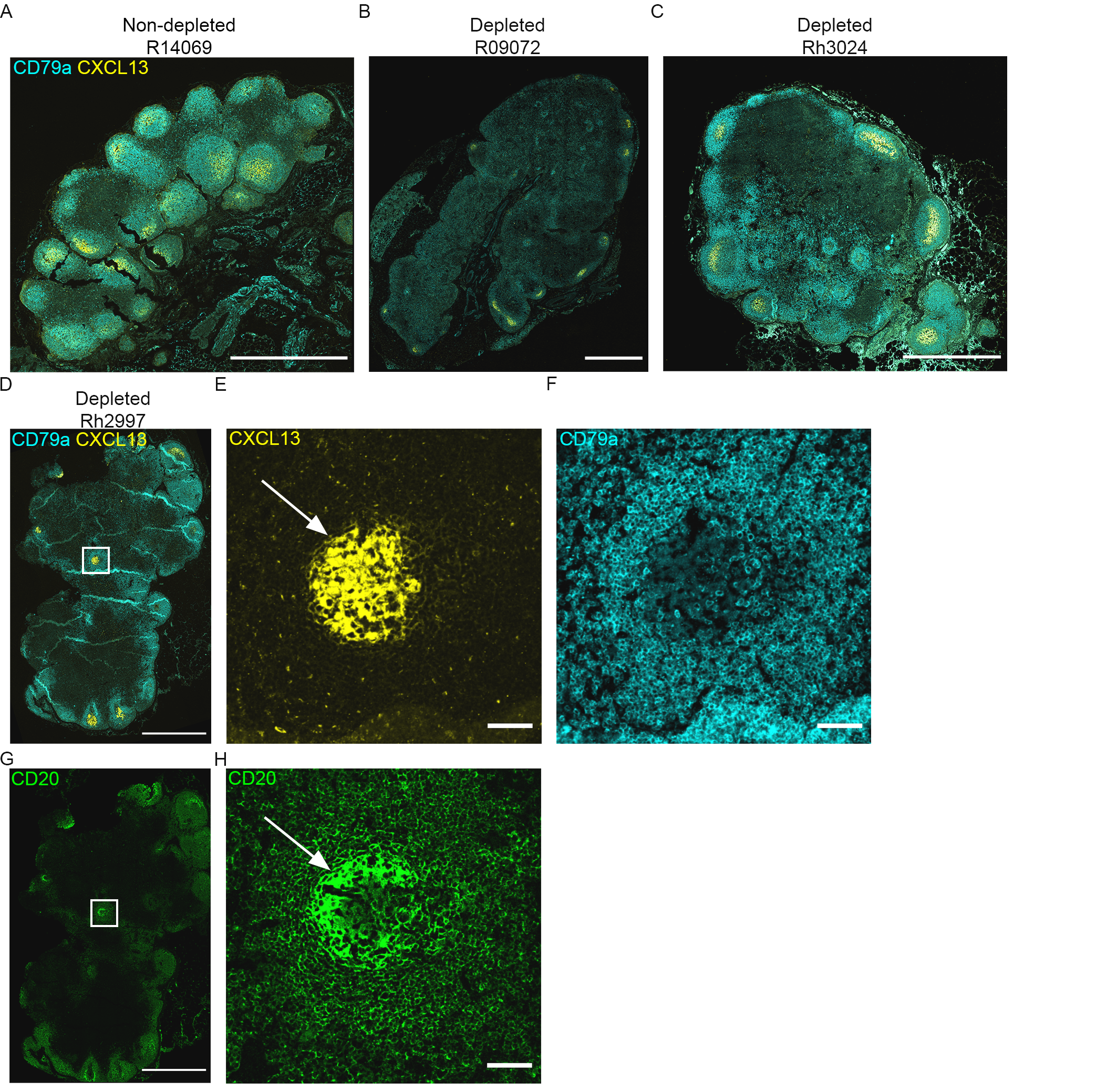

Supplement: Supplementary Figure 3 — B cells and CXCL-13 producing cells detected in lymph node sections with anti-CD79a (cyan) and anti-CXCL13 (yellow) staining at 9 days post-depletion in (A) CAR-T (non-depleted) animal R14069, (B) depleted/CAR-T animal R09072, (C) depleted animal Rh3024, and (D) depleted/CAR-T animal Rh2997. (E, F) are enlargements of the delineated area in (D) showing anti-CXCL13 (E) and anti-CD79a staining (F). The arrow in (E) indicates FDC stained with anti-CXCL13. (G) shows anti-CD20 staining in a section near the section from (D) and (H) is an enlargement of the delineated area in (G). The arrow in (H) indicates CD20 captured on FDC. Scale bars are 1000 µm for (A–D, G) and 50 µm for (E, F, H) [file Image_3.tif]

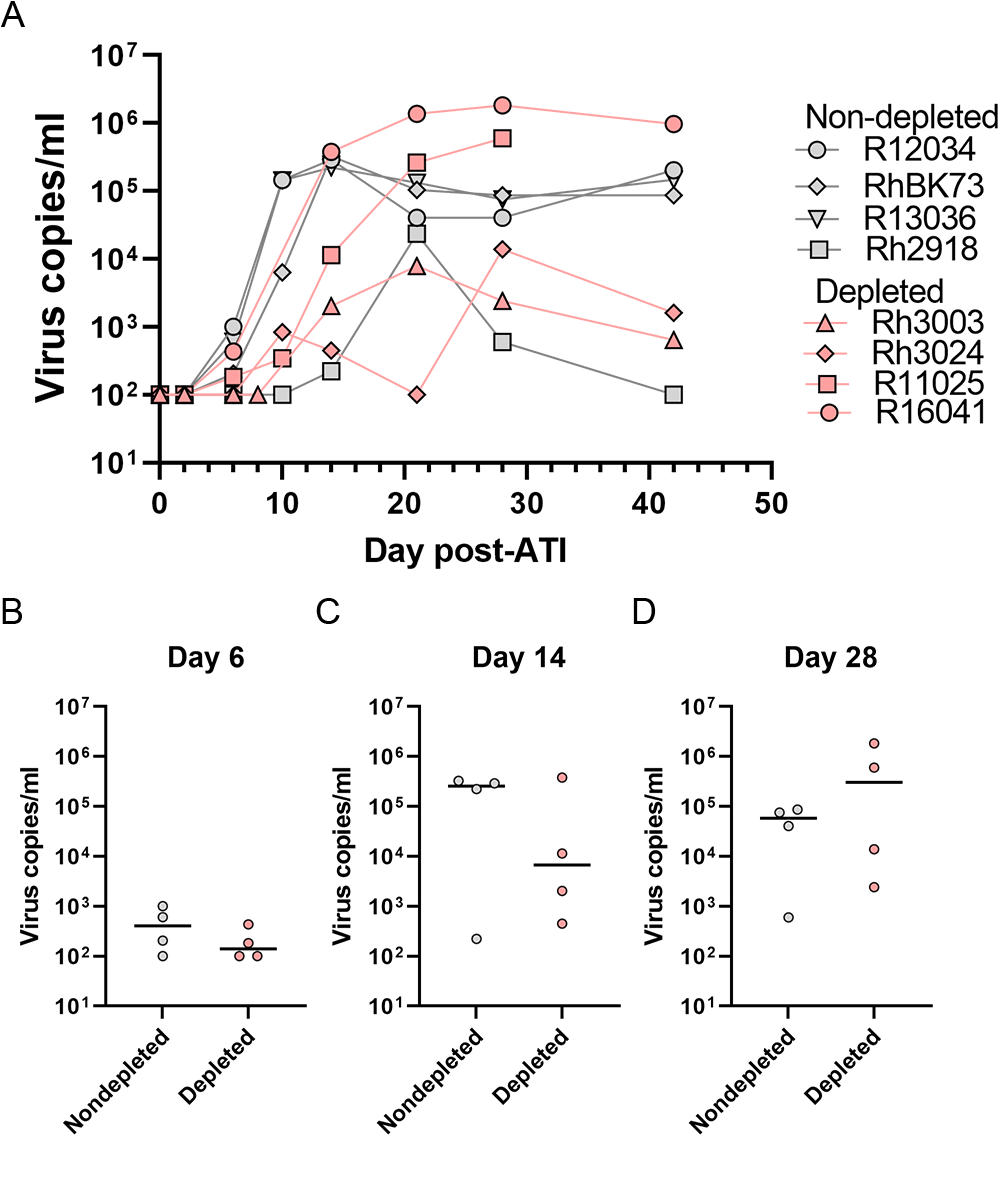

Supplement: Supplementary Figure 4 — Comparison of viral loads over time. (A) Viral loads are presented over time after ART interruption in the non depleted (light gray) and depleted (peach) control animals. Comparison of viral loads at (B) 6 days, (C) 14 days and (D) 28 days post ART interruption. The bars represent the median values for each data set. [file Image_4.tif]
